# Effects of Vericiguat on the Mitochondrial Function and Clinical Outcomes in CRT Nonresponders Patients

**DOI:** 10.1016/j.jacadv.2026.102708

**Published:** 2026-04-04

**Authors:** Celestino Sardu, Nunzia D’Onofrio, Carlo Fumagalli, Maria Luisa Balestrieri, Alessandro Landolfi, Carmine La Marca, Valerio Giordano, Isabella Donisi, Pietro Rambaldi, Gianluca Gatta, Salvatore Cappabianca, Ferdinando Carlo Sasso, Michelangela Barbieri, Giuseppe Paolisso, Raffaele Marfella

**Affiliations:** a Department of Advanced Medical and Surgical Sciences, University of Campania Luigi Vanvitelli, Naples, Italy; b Department of Neuro-Cardiovascular Sciences, IRCCS Neuromed, Pozzilli, Italy; c Department of Experimental Medicine, University of Campania Luigi Vanvitelli, Naples, Italy; d Research Center for Environmental Pollution and Cardiovascular Diseases, University of Campania Luigi Vanvitelli, Naples, Italy; e Department of Cardiovascular Sciences, Santa Maria Della Pietà Hospital, Nola, Naples, Italy; f Department of Cardiovascular Sciences, San Luca Hospital Hospital, Vallo Della Lucania, Italy; g Department of Precision Medicine, University of Campania Luigi Vanvitelli, Naples, Italy; h School of Medicine, “Saint Camillus University”, Rome, Italy

**Keywords:** CRT nonresponders, inflammation, MIBI, mitochondrial function, oxidative stress, vericiguat

## Abstract

**Background:**

Cardiac resynchronization therapy (CRT) failure leads to adverse remodeling and poor outcomes.

**Objectives:**

The purpose of this study was to evaluate vericiguat effects on outcomes, mitochondrial function, and inflammation/oxidative stress in CRT nonresponders at 1 year.

**Methods:**

In this multicenter, observational prospective study, CRT nonresponders treated with vericiguat (n = 156) were compared with non-treated (n = 415). Mitochondrial function was assessed by technetium-99m-methoxyisobutylisonitrile uptake and metabolic profiling of peripheral blood mononuclear cells. Primary endpoint was the CRT responder rate at 1 year; secondary endpoints included heart failure (HF) hospitalizations, mitochondrial function, and inflammatory/oxidative stress markers. CRT responders showed ≥10% reduction in left ventricle end-systolic volume with improved functional status at follow-up. Statistical analysis included Student’s *t*-tests, chi-square tests, Fisher’s tests, linear mixed models, and Cox regression (significance *P* < 0.05).

**Results:**

At 1 year, vericiguat users vs those untreated had higher CRT responders’ rate [67/156 (42.9%) vs 51/415 (12.3%)] and lower HF hospitalizations [17/156 (11.1%) vs 114/415 (27.5%)], (*P* < 0.05). They had better NYHA functional class, higher left ventricular ejection fraction, and lower inflammatory/oxidative stress levels (*P* < 0.05). Reduced methoxyisobutylisonitrile washout, enhanced adenosine triphosphate (ATP) synthesis, and increased sirtuins3-6 expression were observed (*P* < 0.05). CRT-responders outcome was predicted by vericiguat (2.165; 95% CI: 1.512-4.623), sacubitril/valsartan (1.432; 95% CI: 1.020-2.885), and low-baseline ATP (0.265; 95% CI: 0.072-0.369); HF hospitalizations outcome was reduced by vericiguat (0.312; 95% CI: 0.195-0.497) and sacubitril/valsartan (0.672; 95% CI: 0.308-0.865) and increased by lower baseline ATP production (4.881; 95% CI: 1.945-8.507) and left ventricular ejection fraction (1.864; 95% CI: 1.209-2.871) at follow-up end.

**Conclusions:**

In this small cohort of CRT nonresponders, vericiguat increased CRT response at 1 year, likely through improved mitochondrial function and reduced inflammatory/oxidative stress. Larger studies are needed to confirm these findings.

Cardiac resynchronization therapy (CRT) improves functional capacity and quality of life in patients with heart failure (HF) and reduces hospitalizations for HF worsening and mortality.^1^ These ameliorative effects are mainly due to left ventricular (LV) remodeling and an increase in LV ejection fraction (LVEF).^2^

These echocardiographic and clinical effects are observed in approximately two-thirds of treated patients, referred to as CRT responders.^2^ In contrast, CRT failure is driven by complex cellular mechanisms, with mitochondrial dysfunction leading to impaired fatty acid oxidation and reduced adenosine triphosphate (ATP) production.^3^ In this setting, vericiguat is a novel anti-HF drug that could modulate mitochondrial function, thereby restoring impaired nitric oxide (NO), soluble guanylate cyclase (sGC), and cyclic guanosine monophosphate (cGMP) signaling pathways in patients with HF.^4^

In a rat model, vericiguat preserved cardiac function and remodeling, improved mitochondrial quality by reducing reactive oxygen species production and mitochondrial depolarization, and attenuated myocardial fibrosis.^5^ In pigs, vericiguat enhanced myocardial vascularization by reducing inflammation and collagen deposition.^6^ Thus, we hypothesized that vericiguat could improve mitochondrial function and reduce inflammatory/oxidative stress, as well as cardiac remodeling, in patients with CRT failure. These effects may determine the best clinical outcomes at 1-year follow-up. Therefore, we evaluated the impact of vericiguat on mitochondrial function, inflammatory/oxidative stress, and clinical outcomes in patients with CRT nonresponders who received vericiguat (vericiguat users) compared with those who did not (non-vericiguat users) at 1 year of follow-up.

The clinical outcomes investigated were the CRT response (responders’ rate) and the HF hospitalizations at 1 year of follow-up.

## Methods

### Study population

We conducted a multicenter observational prospective study to evaluate the effects of vericiguat on a population of nonresponders to CRT. These HF patients had undergone CRT but failed to meet the following criteria 6 months after device implantation: LV reverse remodeling (reduction in LV end-systolic volume [LVESV] of ≥10% at cardiac echography), significant change in functional HF class (improvement of the 6 minute-walk test improvement and Minnesota living with HF scale improvement).^1^ The study population respected the following inclusion/exclusion criteria:

#### Inclusion criteria

At least 18 years of age, with a clinical history of stable chronic HF, NYHA functional class II, III, or IV, left bundle branch block, severe left ventricle ejection fraction reduction (LVEF <35%), stable sinus rhythm, and defined CRT nonresponders according to the diagnostic criteria;^1^ patients with an estimated glomerular filtration rate of at least 30 mL/min/1.73 m^2^ of the body surface area.

#### Exclusion criteria

Age <18 or >75 years, ejection fraction >40%, diagnosis of CRTd responders, unstable HF, patients in IV NYHA functional class, hyperkalemia, systolic hypotension (systolic blood pressure <90 mm Hg); patients with an estimated glomerular filtration rate <30 mL/min/1.73 m^2^ of the body surface area; absence of informed patient consent, and any condition that would make survival for 1 year unlikely. Thus, we enrolled a consecutive population of CRTd nonresponders with HF.

Vericiguat has class IIb evidence according to the latest American Heart Association HF guidelines.^4^ CRT nonresponders in NYHA functional class II-IV with recent HF hospitalization despite optimal medical therapy, and high risk of having HF worsening, were treated with vericiguat. The risk of HF worsening was defined as an elevation of B-type natriuretic peptides or N-terminal pro-B-type natriuretic peptides (BNP ≥300 ng/L or NT-proBNP ≥1,000 ng/L in patients in sinus rhythm; BNP ≥500 ng/L or NT-proBNP ≥1,600 ng/L in patients in atrial fibrillation) and the presence of a HF hospitalization in the previous 6 months or intravenous diuretic therapy in the last 3 months.^7^ Vericiguat was introduced into clinical practice in June 2023.

Furthermore, we had patients who did not receive vericiguat, represented by CRT nonresponders enrolled before June 2023 and defined as vericiguat nonusers.

The CRT nonresponders enrolled after July 2023 and treated with vericiguat were defined as vericiguat users and were not randomized to vericiguat therapy in our study. Vericiguat users were selected as patients who were under treatment before the start of the study. The study cohorts were followed for 1 year. For vericiguat users, the 1-year follow-up was calculated starting from the initiation of vericiguat therapy. For non-vericiguat users, time 0 corresponded to the baseline clinical evaluation performed at the same stage of CRT nonresponders' diagnosis. Thus, both cohorts were followed prospectively for 12 months from their respective baseline assessments, ensuring comparable follow-up intervals across groups.

### Study design

In a prospective observational multicenter study conducted from June 2018 to November 2023, we enrolled CRT nonresponders and divided into 2 cohorts: 1) CRT nonresponders who received vericiguat (vericiguat users); and 2) CRT nonresponders who did not receive vericiguat (non-vericiguat users). The patients enrolled in the study were evaluated using clinical and echocardiographic assessments, as well as telemetric interrogation of the CRT device at baseline and at 6 and 12 months of follow-up. This procedure allows the assessment of CRT pacing effectiveness, detection of arrhythmic events, and monitoring of battery and lead integrity, as well as documentation of device-delivered therapies such as shocks or other CRT interventions. We reported the CRT effect on clinical and echocardiographic parameters during quarterly visits, as well as the CRT responder rate. The study was conducted in accordance with the Declaration of Helsinki, and the Ethics Committee approved the protocol, which was registered on the public registry (NCT03282136). All patients were informed about the study’s nature and signed informed consent to participate. All patients underwent clinical, hematological, and instrumental evaluations.

### Anthropometric evaluations

At baseline and at follow-up, we evaluated the physical examination and vital signs in the CRT nonresponders and selectively in vericiguat users vs vericiguat nonusers. The body mass index was calculated as the ratio between weight in kg and the height squared.^8^ The clinical evaluations included physical examinations, vital signs, and the review of adverse events at baseline and at follow-up.

### Echocardiographic evaluation

Two physicians, blinded to the study protocol and experienced in transthoracic 2-dimensional echocardiography, performed cardiac echography at baseline and follow-up, including M-mode recordings, conventional Doppler, and pulsed-wave tissue Doppler imaging measurements, using a Philips iE33 echocardiography system (Eindhoven, The Netherlands). The echocardiographic images were acquired in the parasternal long- and short-axis views. The LV end-diastolic diameter, LV end-diastolic volume (LVEDV), LV end-systolic diameter, and LVESV were measured, and the LVEF was calculated by the Simpson method.^9^ The amount of mitral regurgitation was calculated as the area of the color-flow Doppler regurgitant jet divided by the area of the left atrium in systole and described as mild, moderate, moderate-severe, and severe, as previously reported.^9^ The echocardiographic measurements in 5 consecutive samples were systematically averaged.

### Laboratory analysis

Peripheral venous blood samples were collected after an overnight fast at baseline and follow-up to measure plasma glucose, serum lipids, BNP, and NT-proBNP using enzymatic assays. Samples were processed immediately, centrifuged, and stored at −80 °C. Inflammatory and oxidative stress markers were measured in duplicate by using a highly sensitive quantitative sandwich enzyme-linked immunosorbent assay (ELISA, Quantikine HS; R&D Systems). Inflammatory molecular and cellular markers included circulating serum levels of pro-inflammatory cytokines (tumor necrosis factor [TNF]-α, interleukin [IL]-6), inflammatory markers (C-reactive protein [CRP]), leukocyte and neutrophil counts. Oxidative stress was assessed by serum nitrotyrosine levels by ELISA. See the Supplemental Appendix.

### Peripheral blood mononuclear cell isolation

Peripheral blood (10 mL) was collected from vericiguat-treated and untreated patients in heparinized tubes and processed within 2 hours. Peripheral blood mononuclear cells (PBMCs) were isolated by density gradient centrifugation using Histopaque-1,077 (10,771, Sigma), washed twice with phosphate-buffered saline, resuspended in RPMI 1640 medium (L0500, Aurogene) supplemented with 10% fetal bovine serum, and incubated at 37 °C with 5% CO_2_. Cells were used for assessment of mitochondrial bioenergetics, including oxygen consumption rate (OCR), ATP production, proton leak, glycolytic flux, and cytokine secretion profiles.^10^ See the Supplemental Appendix.

### Metabolic analysis

Cellular bioenergetics were assessed using the Seahorse XF HS Mini Analyzer (Agilent Technologies). Mitochondrial respiration and glycolysis were measured using the Seahorse Cell Mito Stress Test (103010-100, Agilent) and Glycolysis Stress Test (103020-100, Agilent), respectively. PBMCs were seeded at 2 × 10^4^ cells/well in XF DMEM and incubated before analysis. Sequential injections of oligomycin, FCCP, and rotenone/antimycin A assessed mitochondrial respiration, while glucose, oligomycin, and 2-deoxy-D-glucose assessed glycolytic flux. Data were normalized to protein content per well. See the Supplemental Appendix.

### Glycolytic function assay

Glycolytic flux in PBMCs was assessed by extracellular acidification rate (ECAR) using the Seahorse XF HS Mini Analyzer (Agilent Technologies). Cells were sequentially exposed to glucose, oligomycin, and 2-deoxyglucose to quantify basal glycolysis, glycolytic capacity, and glycolytic reserve. ECAR values were normalized and analyzed at baseline and follow-up in vericiguat-treated and untreated patients. See the Supplemental Appendix.

### Sirtuins3 enzymatic activity assay

Sirtuins (SIRT)3 enzymatic activity was measured using a fluorometric assay kit (BML-AK557, Enzo Life Sciences). Equal amounts of protein were incubated with an acetylated fluorogenic substrate in the presence of NAD^+^, and fluorescence was quantified at 360/460 nm. SIRT3 activity was expressed as a percentage relative to non-vericiguat users, set as 100%. See the Supplemental Appendix.

### Immunoblotting analysis

The immunoblotting analysis was used to quantify the expression of mitochondrial SIRT (SIRT3, SIRT4, SIRT5), SIRT6, and the inflammasome protein NLRP3, as mechanistic markers of mitochondrial function, oxidative stress, and inflammatory signaling.^11^^,^^12^ Protein expression was analyzed by Western blotting. Cell lysates were prepared in RIPA buffer with protease and phosphatase inhibitors, and equal amounts of protein were separated by sodium dodecyl sulfate-polyacrylamide gel electrophoresis and transferred to nitrocellulose membranes. Membranes were probed with antibodies against SIRT3, SIRT4, SIRT5, SIRT6, and NLRP3, followed by HRP-conjugated secondary antibodies. Immunoreactive bands were detected by enhanced chemiluminescence, quantified using ImageJ, and normalized to loading controls. See the Supplemental Appendix.

### ELISA assays

After isolation, cells were seeded at 1 × 10^6^ cells/well and incubated for 24 hours at 37 °C with 5% CO_2_. Supernatants were collected, centrifuged, and analyzed for cytokine secretion including interferon gamma (ab174443, Abcam), TNF-α (ab181421, Abcam), IL-6 (ELK1156, ELK Biotechnology), IL-18 (ab215539, Abcam), IL-1β (ab214025, Abcam), IL-10 (ab174443), and IL-8 (ab46032, Abcam), measured using ELISA kits specific for human targets. Absorbance was read at 450 nm, and cytokine concentrations were calculated from standard curves. See the Supplemental Appendix.

### Quantitative reverse-transcription polymerase chain reaction analysis

Total RNA was isolated from PBMC pellets using Trizol reagent according to the manufacturer’s procedures. RNA integrity was assessed by the NanoDrop 2000c spectrophotometer (Thermo Scientific). The expression of SIRT3, SIRT4, SIRT5, and SIRT6 genes was evaluated in PBMCs by quantitative reverse-transcription polymerase chain reaction by using ACTB (β-actin) as housekeeping gene, according to the protocol previously described by Owczarz et al.^12^ Results were quantified by using the 2^−ΔΔCt^ method.

### Cardiac scintigraphy with methoxyisobutylisonitrile

Mitochondrial function was analyzed in the study population by visualizing and evaluating cardiac scintigraphy accumulation of technetium-99m methoxyisobutylisonitrile (MIBI) at baseline and at follow-up. The exams were performed at baseline and follow-up end in the CRT nonresponders. We used MIBI (600 MBq; Daiichi Radioisotope Laboratories Ltd), injected intravenously via peripheral vein access, and obtained myocardial images with a standard-field gamma camera equipped with a low-energy, general-purpose collimator (Ecam Dual Head, Siemens). The images obtained 1 hour after the injection were reviewed as the early images, and subsequently delayed images at 2-hour intervals.^3^^,^^10^, ^11^, ^12^, ^13^ We acquired the MIBI planar images (512 × 512 pixels) of the anterior thorax and the MIBI single-photon emission computed tomography data obtained over 180° in 60 steps, each lasting 30 seconds. From the images, we calculated LVEDV, LVESV, and LVEF using quantitative electrocardiography-gated single-photon emission computed tomography programs.^10^ The tracer uptake was quantified using processing equipment (GMS 5500 A/DI; Toshiba). The 99mTC-sestamibi myocardial wash-out was measured at baseline and 12 months’ follow-up to assess the effect of vericiguat vs conventional anti-HF therapy on in vivo mitochondrial function in CRT nonresponders. The full description is reported in the Supplemental Appendix.

### Study endpoints

We evaluated, in CRT nonresponders patients divided into vericiguat users vs non-vericiguat users, the following primary and secondary study endpoints at 1 year.

The primary outcome of the study was the response to CRT (number of CRT responders) at 1-year follow-up. CRT responders were defined the patients with evidence of ≥10% reduction in LVESV, and improvement in functional status (NYHA class: ≥1 class improvement, 6MWT: ≥30-m increase, Global self-assessment [patient-reported classification of improved vs unchanged/worsened using a structured ordinal scale]), consistent with established criteria.^1^, ^2^, ^3^, ^4^ At least one functional improvement criterion, plus LV reverse remodeling, was required for diagnosis of CRT responders.^1^, ^2^, ^3^, ^4^

The secondary outcomes included: 1) HF hospitalizations at 1 year; 2) the mitochondrial function, inflammatory oxidative stress burden and molecular endpoints. Mitochondrial function was assessed by bioenergetic profiling of PBMCs, including OCR, ATP production, proton leak, and glycolytic flux, and cardiac mitochondrial function evaluated by technetium-99m MIBI washout rate and heart-to- mediastinum ratio;^11^, ^12^, ^13^ 3) inflammatory and oxidative stress burden was measured by circulating cytokines (TNF-α, IL-6, IL-1β, IL-18, IL-8, IL-10), CRP, nitrotyrosine, leukocyte counts, and PBMC expression of NLRP3. The molecular endpoints included SIRT3, SIRT4, SIRT5, and SIRT6 protein and mRNA expression, and SIRT3 enzymatic activity, evaluated as mechanistic markers of mitochondrial adaptation. We also examined other clinical parameters (NYHA functional class and 6MWT distance) and echocardiographic parameters (LVEF, LV volumes [LVEDV, LVESV], LV diameters, and the severity of mitral regurgitation). NYHA functional class was re-evaluated for each patient at follow-up. The patients graded their overall condition as unchanged, slightly worsened, moderately worsened, markedly worsened, or improved using a global self-assessment.^1^, ^2^, ^3^, ^4^ We instructed all the patients to assess body weight, the occurrence of dyspnea, and any clinical symptoms.

### Statistical methods

A qualified statistician analyzed all the collected data. CRTd nonresponders were divided into the vericiguat users vs the nonusers’ patients. We calculated that the number of patients with alterations in the study endpoints differed significantly between the 2 study cohorts. The safety analysis was performed on data from all enrolled patients. Given the observational nature of this study, safety analysis was focused on clinically relevant adverse events, including symptomatic hypotension, worsening renal function, electrolyte abnormalities, and treatment discontinuation. These outcomes were assessed through a systematic review of medical records and laboratory data during follow-up and compared between treatment groups.

Continuous variables were expressed as means and SDs and tested by a 2-tailed Student’s *t*-test. We compared categorical variables using the chi-square or Fisher’s exact test, as appropriate.

To evaluate longitudinal changes in the study variables, echocardiographic parameters, biomarkers of inflammation, medications, bioenergetic profile of PBMCs, mitochondrial respiration, SIRT expression, and cytokine secretion and inflammasome-related signaling in vericiguat users vs nonusers, the data collected at baseline and at 1-year follow-up were analyzed using linear mixed models (LMMs) for repeated measures, with time considered as a within-subject factor.

LMMs analyzed the longitudinal changes within and between groups, with time as a within-subject factor and treatment group as a between-subject factor. The group × time interaction was tested to evaluate whether trajectories of change differed between groups. In the Supplemental Appendix, we reported the description of LMMs analysis.

Alternatively, when appropriate, a 2-way analysis of variance including time as a factor was performed. Group differences and time × group interactions were reported.

Predictors of the response to CRT and HF hospitalizations (in the cohorts of CRT nonresponders) were evaluated using Cox regression models in the study population adjusted for study variables: age, II NYHA class, 6MWT, diabetes mellitus, hypertension, BNP, lymphocytes/neutrophils ratio, CRP, ATP production, LVEF, vericiguat, and angiotensin receptor-neprilysin inhibitor therapy. The proportional hazards assumption for all Cox regression models was evaluated by inspecting Schoenfeld residuals and testing for time-dependent covariates. No significant violations of the proportional hazards’ assumption were observed, confirming the validity of the Cox regression models.

As a sensitivity analysis, a Poisson regression model was used to evaluate CRT responder and HF hospitalization rates at 12 months. See the Supplemental Appendix.

Kaplan-Meier curves were generated to evaluate time-to-event outcomes, and the cumulative risk of study outcomes (CRT responders and HF hospitalizations) at 1-year follow-up was assessed. We evaluated the between-group differences using the log-rank test. Statistical significance was considered for a *P* value of < 0.05. The statistical analysis was performed using SPSS software package for Windows 23.0 (SPSS 23 Inc).

## Results

We presented the clinical data at baseline and at 1 year, as well as the results of the primary and secondary study endpoints in the vericiguat users vs nonusers at 1 year.

### Clinical data

The clinical characteristics of vericiguat users vs non-vericiguat users at baseline are reported in Table 1. At baseline, the study cohorts did not differ in clinical and echocardiographic characteristics or in drug therapy (*P* > 0.05) (Table 1). At baseline, we did not find significant differences in inflammatory/oxidative stress markers (*P* > 0.05) (Table 1). In our study, the patients received an initial dose of 2.5 mg once daily, which was titrated to 5 mg and then 10 mg once daily as tolerated, with the target maintenance dose being 10 mg once daily.^10^ The mean dose of vericiguat was 7.9 ± 1.8 mg/day, and 101/156 (65%) patients reached the target dose of 10 mg/day, while 37/156 (24%) were maintained on 5 mg/day, and 18/156 (11%) remained at 2.5 mg/day. The median duration of therapy was 11.9 months (IQR: 11.2-12.4 months). The treatment continued for the entire 12-month follow-up period.

**Table 1:** Clinical Characteristics of Study Population at Baseline and at 1 Year of Follow-Up

	At Baseline			At 1 Year of Follow-Up		
	Vericiguat-Users (n = 156)	Non-Vericiguat Users (n = 415)	*P* Value	Vericiguat-Users (n = 123)	Non-Vericiguat Users (n = 301)	*P* Value
Age, y	71.4 ± 4.6	71.5 ± 6.4	0.771	/	/	/
Male, n (%)	96 (61.5)	289 (69.6)	0.066	/	/	/
BMI >30 kg/m^2^, n (%)	12 (7.7)	29 (7.0)	0.914	8 (6.5)	26 (8.6)	0.330
Smokers, n (%)	85 (54.5)	237 (57.1)	0.640	73 (59.3)	181 (60.1)	0.281
Hypertension, n (%)	110 (70.5)	311 (74.9)	0.335	90 (73.2)	241 (80.1)	0.086
Diabetes mellitus, n (%)	109 (69.9)	270 (65.1)	0.258	89 (72.4)	202 (67.1)	0.212
Dyslipidemia, n (%)	76 (48.7)	209 (50.4)	0.798	55 (44.7)	138 (45.8)	0.740
COPD, n (%)	41 (26.3)	115 (27.7)	0.813	34 (27.6)	87 (28.9)	0.830
IDCM, n (%)	93 (59.6)	235 (56.6)	0.583			
NYHA functional class I, n (%)	/	/	0.619	4 (3.2)	/	0.001^a^
NYHA functional class II, n (%)	65 (41.7)	166 (40.0)		67 (54.5)	81 (26.9)	
NYHA functional class III, n (%)	73 (46.8)	206 (49.6)		46 (37.4)	184 (61.1)	
NYHA functional class IV, n (%)	18 (11.5)	43 (10.4)		6 (4.9)	36 (12)	
QRS duration, ms	135.8 ± 8.0	136.1 ± 7.2	0.686	142.5 ± 8.0	142.8 ± 6.9	0.674
6MWT	189.78 ± 26.95	193.24 ± 19.42	0.144	252.25 ± 43.57	213.93 ± 25.42	0.002^a^
Systolic blood pressure, mm Hg	115.4 ± 6.3	116.1 ± 7.1	0.412	117.2 ± 8.7	118.5 ± 9.5	0.503
Heart rate (beats/min)	72.1 ± 6.6	71.7 ± 7.0	0.601	70.4 ± 6.1	71.0 ± 6.3	0.447
Echocardiographic parameters						
LVEF (%)	26.1 ± 8.7	27.0 ± 10.5	0.765	39.3 ± 4.6	31.7 ± 4.5	0.001^a^
LVEDd (mm)	65.3 ± 7.6	64.5 ± 6.8	0.376	59.9 ± 8.3	62.0 ± 7.1	0.027^a^
LVESd (mm)	41.3 ± 4.9	40.9 ± 5.9	0.474	37.5 ± 5.2	39.2 ± 5.9	0.005^a^
LVEDv (mL)	220.1 ± 22.1	217.2 ± 15.6	0.136	172.1 ± 27.2	198.7 ± 19.9	0.002^a^
LVESv (mL)	135.5 ± 17.1	133.0 ± 18.4	0.142	105.8 ± 33.8	125.4 ± 22.2	0.001^a^
Mitral insufficiency			0.688			0.05^a^
Mild (%)	58 (37.2)	169 (40.7)		64 (52.0)	91 (30.2)	
Moderate (%)	76 (48.7)	205 (49.4)		52 (42.3)	192 (63.8)	
Moderate-severe (%)	22 (14.1)	41 (9.9)		7 (5.7)	18 (6)	
Biomarkers of inflammation						
Lymphocytes, n × 10^3^	6.94 ± 1.18	7.14 ± 1.79	0.212	6.12 ± 1.30	7.97 ± 1.52	0.001^a^
Neutrophils, n	4.66 ± 0.98	4.74 ± 0.89	0.346	5.30 ± 1.38	5.78 ± 1.20	0.001^a^
LNR	1.54 ± 0.37	1.56 ± 0.48	0.693	1.21 ± 0.35	1.43 ± 0.39	0.001^a^
BNP, (pg/mL)	341.9 ± 187.8	322.6 ± 201	0.297	166.52 ± 86.1	353.6 ± 150.3	0.001^a^
NT-proBNP, (pg/mL)	1836.8 ± 958.6	1728.7 ± 706.2	0.147	1,056.9 ± 108.6	1,468.8 ± 93.6	0.001^a^
CRP (mg/L)	6.09 ± 0.49	5.72 ± 0.39	0.387	6.98 ± 0.49	8.93 ± 0.30	0.001^a^
Nitrotyrosine (A.U.)	51.50 ± 4.45	51.10 ± 4.74	0.359	36.03 ± 12.49	45.27 ± 11.06	0.001^a^
Creatinine (md/dL)	1.15 ± 0.32	1.17 ± 0.34	0.672	1.13 ± 0.30	1.16 ± 0.33	0.588
Medications						
Amiodarone, n (%)	37 (23.7)	109 (26.3)	0.534	26 (21.1)	87 (28.9)	0.088
ACE inhibitors, n (%)	58 (37.2)	184 (44.3)	0.123	40 (32.5)	123 (40.9)	0.088
ARS blockers, n (%)	25 (16)	81 (19.5)	0.339	17 (13.8)	51 (16.9)	0.391
Sacubitril/valsartan, n (%)	35 (22.4)	103 (24.8)	0.553	34 (27.6)	111 (36.9)	0.042^a^
Beta-blockers						
Carvedilol, n (%)	72 (46.2)	189 (45.5)	0.765	59 (48.0)	142 (47.2)	0.857
Bisoprolol, n (%)	52 (33.3)	132 (31.8)	0.728	36 (29.53)	97 (32.2)	0.557
Aspirin, n (%)	58 (37.2)	178 (42.9)	0.217	54 (43.9)	144 (47.8)	0.417
Tiklopidine, n (%)	5 (3.2)	18 (4.3)	0.540	7 (5.7)	18 (6)	0.909
Warfarin, n (%)	35 (22.4)	90 (21.7)	0.847	31 (25.2)	79 (26.2)	0.759
NOAC, n (%)	48 (30.8)	108 (26)	0.257	39 (31.7)	91 (30.2)	0.809
Calcium antagonist, n (%)	10 (6.4)	36 (8.7)	0.376	10 (8.1)	35 (11.6)	0.265
Ivabradine, n (%)	38 (24.4)	109 (26.3)	0.643	35 (28.5)	92 (32.9)	0.341
Digoxin, n (%)	60 (38.4)	148 (35.7)	0.536	44 (35.8)	109 (36.2)	0.802
Loop diuretics, n (%)	134 (85.9)	364 (87.7)	0.563	101 (82.1)	277 (92.0)	0.001^a^
Aldosterone blockers, n (%)	91 (58.3)	235 (56.6)	0.713	66 (53.7)	184 (61.1)	0.123
SGLT2i, n (%)	25 (16)	74 (17.8)	0.612	25 (20.3)	99 (32.9)	0.005^a^
Statins, n (%)	115 (73.7)	308 (74.2)	0.904	95 (77.2)	240 (79.7)	0.459
Insulin, n (%)	37 (23.7)	95 (20.9)	0.835	35 (28.4)	73 (24.2)	0.344
Metformin, n (%)	88 (56.4)	246 (59.3)	0.565	72 (58.5)	187 (62.1)	0.465
Sulfonylureas, n (%)	36 (23.1)	89 (21.4)	0.674	34 (27.6)	69 (22.9)	0.272
Thiazolidinediones, n (%)	17 (10.9)	50 (12)	0.703	16 (13.0)	42 (13.9)	0.776
GLP-1 agonist, n (%)	26 (16.7)	69 (16.6)	0.991	23 (18.7)	60 (19.9)	0.802
DPP-4 inhibitors, n (%)	30 (19.2)	86 (20.7)	0.683	28 (22.8)	70 (23.3)	0.860

ACE = angiotensin-converting enzyme; ARS = angiotensin receptors blockers; A.U. = arbitrary units; BMI = body mass index; BNP = B-type natriuretic peptide; COPD = chronic obstructive pulmonary disease; CRP = C-reactive protein; DPP-4 = dipeptidyl peptidase 4; GLP-1 = glucagon-like peptide-1; IDCM = ischemic dilated cardiomyopathy; LNR = lymphocytes/neutrophils ratio; LVEDd = left ventricle end-diastolic diameter; LVEDv = left ventricle end-diastolic volume; LVEF = left ventricle ejection fraction; LVESd = left ventricle end-systolic diameter; LVESv = left ventricle end-systolic volume; 6MWT = 6 minutes walking test; NOAC = new oral anticoagulants; NT-proBNP = N-terminal pro-B-type natriuretic peptide; SGLT2i = sodium glucose co-transporter 2 inhibitors.

a Statistically significant (*P* < 0.05). *P* values represent comparisons between vericiguat-users and vericiguat nonusers at baseline and at follow-up end.

At 1 year of follow-up, a higher percentage of vericiguat users vs non-vericiguat users were in I (4/123 [3.2%] vericiguat users vs 0/301 [0%] nonusers) and II NYHA functional class (67/123 [54.5%] vs 81/301 [26.9%]) (*P* < 0.05), and a lower rate of vericiguat users vs non-vericiguat users were in III (46/123 [37.4%] vs 184/301 [61.1%]) and IV NYHA functional class (6/123 [4.9%] vs 36/301 [12.0%]) (*P* < 0.05); vericiguat users vs nonusers showed a significant increase of 6MWT (*P* < 0.05) (Table 1).

Regarding echocardiographic parameters, at 1 year of follow-up, vericiguat users vs nonusers showed a significant reduction in LV diastolic and systolic diameters/volumes and of the degree of mitral valve insufficiency, and a substantial increase in LVEF (*P* < 0.05) (Table 1). We noted a significant reduction of inflammatory cells/molecules and nitrotyrosine in vericiguat users vs non-vericiguat users at 1 year of follow-up (*P* < 0.05) (Table 1). Finally, vericiguat users vs nonusers showed a lower rate of patients under anti-HF drugs (sacubitril/valsartan: 34/123 [27.6%] vs 111 [36.9%]; loop diuretics: 101/123 [82.1%] vs 277/123 [92.0%]; and sodium glucose co-transporter 2 inhibitors: 25/123 [20.3%] vs 99/301 [32.9%]) at 1 year of follow-up (*P*< 0.05) (Table 1). Within-group analyses showed that vericiguat users had significant improvements from baseline in LVEF, LVEDV, LVESV, 6MWT, NYHA functional class, and MIBI-WR (*P* < 0.05), whereas nonusers did not show significant changes over time (*P* > 0.05). Mixed-model analyses confirmed significant group × time interactions across these endpoints, indicating that vericiguat therapy was associated with more favorable longitudinal trajectories. The comparison between follow-up end vs baseline data for each cohort of the study is reported in the Supplemental Appendix and the Supplemental Table.

### Clinical outcomes: CRT-responders rate and HF hospitalizations

A higher rate of CRT responders (67/156 [42.9%] vs 51/415 [12.3%]; *P* < 0.05) and a lower rate of HF hospitalizations (17/156 [11.1%] vs 114/415 [27.5%]; *P* < 0.05) were found in the vericiguat users vs nonusers at 1 year of follow-up. The study cohorts did not differ significantly in mortality (cardiac deaths: 21 [13.5%] vs 68 [16.4%], *P* > 0.05; all-cause deaths: 33 [21.2%] vs 114 [27.5%], *P* > 0.05).

For the CRTd responders’ outcome, the Cox regression analysis showed that vericiguat (HR: 2.165; 95% CI: [1.512-4.623]) and sacubitril/valsartan therapy (HR: 1.432; 95% CI: [1.020-2.885]) increased the CRT responder outcome at 1 year of follow-up (*P* < 0.05) (Table 2). On the contrary, the lowest baseline ATP production reduced the CRT responder outcome (HR: 0.265; 95% CI: [0.072-0.369]) at 1 year of follow-up (*P* < 0.05) (Table 2).

**Table 2:** Cox Regression Analysis for Study Endpoint of Cardiac Resynchronization Therapy Responders and Hospitalizations for Heart Failure Worsening at 1 Year of Follow-up in the Study Population of 571 Patients

	Univariate Analysis			Multivariate Analysis		
	HR	95% CI	*P* Value	HR	95% CI	*P* Value
CRT responders						
Age	0.994	0.964-1.024	0.685	0.981	0.942-1.021	0.312
II NYHA	1.191	0.828-1.713	0.346	1.087	0.701-1.684	0.710
6MWT	1.007	0.998-1.015	0.113	1.004	0.996-1.012	0.304
Diabetes mellitus	1.781	1.159-2.737	0.008^a^	1.431	0.873-2.345	0.155
Hypertension	1.752	1.093-2.809	0.200^a^	0.812	0.431-1.529	0.516
BNP	0.999	0.998-1.000	0.021^a^	0.991	0.982-1.004	0.163
LNR	0.983	0.666-1.451	0.933	0.902	0.601-1.354	0.624
CRP	0.998	0956-1.042	0.920	0.991	0.958-1.026	0.648
ATP production	0.180	0.079-0.409	0.001^a^	0.265	0.072-0.369	0.001^a^
LVEF	0.409	0.073-2.297	0.310	0.871	0.315-2.407	0.784
Vericiguat	3.993	2.777-5.751	0.001^a^	2.165	1.512-4.623	0.001^a^
Sacubitril/valsartan	1.623	1.106-2.382	0.013^a^	1.432	1.020-2.885	0.040^a^
HF worsening hospitalizations						
Age	1.009	0.982-1.037	0.521	1.006	0.978-1.034	0.472
II NYHA	0.519	0.356-0.757	0.001^a^	0.601	0.389-1.029	0.121
6MWT	0.993	0.986-1.011	0.107	0.996	0.985-1.007	0.441
Diabetes mellitus	1.278	0.881-1.853	0.196	1.115	0.713-1.744	0.629
Hypertension	1.892	1.209-2.963	0.005^a^	1.447	0.903-2.318	0.121
BNP	0.909	0.898-1.000	0.039^a^	0.932	0.821-1.056	0.249
LNR	2.032	1.383-2.985	0.001^a^	1.614	0.987-2.548	0.140
CRP	0.907	0.853-0.964	0.002^a^	0.941	0.891-1.115	0.132
ATP production	3.457	2.213-5.354	0.001^a^	4.881	1.945-8.507	0.001^a^
LVEF	1.509	2.204-10.339	0.001^a^	1.864	1.209-2.871	0.004^a^
Vericiguat	0.466	0.295-0.736	0.001^a^	0.312	0.195-0.497	0.001^a^
Sacubitril/valsartan	0.265	0.146-0.479	0.001^a^	0.672	0.308-0.865	0.001^a^

ATP = adenosine triphosphate; other abbreviations as in Table 1.

a Statistically significant (*P* < 0.05).

The HF worsening hospitalizations outcome was predicted by the lowest baseline ATP production (HR: 4.881; 95% CI: [1.945-8.507]), LVEF (HR: 1.864; 95% CI: [1.209-2.871]), vericiguat therapy (HR: 0.312; 95% CI: [0.195-0.497]), and sacubitril/valsartan therapy (HR: 0.672; 95% CI: [0.308-0.865]) at 1 year of follow-up (*P* < 0.05) (Table 2).

**Figure 1:**
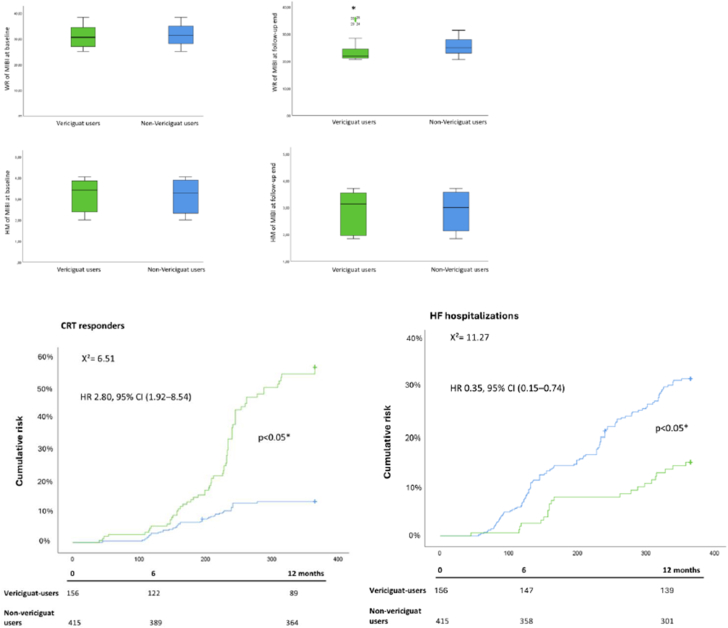
**Representation of WHR and HM by Methoxyisobutylisonitrile Evaluation in the Study Cohorts, and Kaplan-Meier Curves for Study Endpoints in the Study Cohorts at Follow-Up End** In the upper part of the figure, the cardiac accumulation of the technetium 99m methoxyisobutylisonitrile, via the evaluation of wash out rate (WHR) and heart-mediastinum rate (HM) in vericiguat users (green color) compared to non-vericiguat users (blue color), at baseline and follow-up end. In the inferior part of the figure, the Kaplan-Meier curves for the cumulative risk of having the clinical outcomes of cardiac resynchronization therapy responders (left) and heart failure hospitalizations in the vericiguat users (green color) compared to non-vericiguat users (blue color) at 1 year of follow-up. We included in the Kaplan-Meier curves for the study endpoints (cardiac resynchronization therapy responders and heart failure hospitalizations) the HR and CI and number at risk at 0, 6, and 12 months of follow-up for vericiguat-users vs nonusers. ∗Statistical significant (*P* < 0.05). CRT = cardiac resynchronization therapy; HF = heart failure.

The Kaplan-Meier curves indicated a higher risk of having CRT responders and a reduced risk of experiencing HF hospitalization outcomes at 1 year of follow-up among vericiguat users compared to nonusers (Figure 1).

**Figure 2:**
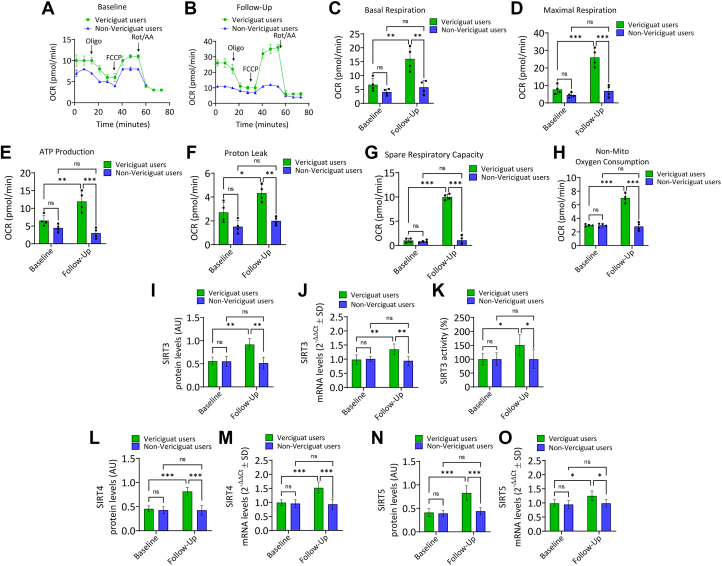
**Vericiguat Improves Mitochondrial Respiration and Upregulates Mitochondrial Sirtuins** In this figure, we evaluated the cellular bioenergetic capacity of vericiguat users (green color) compared to non-vericiguat users (blue color) at baseline and at follow-up. (A, B) The oxygen consumption rate was measured in real-time using the Seahorse XF Mito Stress Test on peripheral blood mononuclear cells isolated from vericiguat-treated and untreated patients. Quantification of (C) basal respiration, (D) maximal respiration, (E) adenosine triphosphate production, (F) proton leak, (G) spare respiratory capacity, and (H) nonmitochondrial oxygen consumption. Immunoblotting analysis and mRNA levels of mitochondrial sirtuins: (I, J) SIRT3, with (K) enzymatic activity expressed as percentage of non-vericiguat users, (L, N) SIRT4, and (N, O) SIRT5. Data are presented as mean ± SD. ∗*P* < 0.05, ∗∗*P* < 0.01, ∗∗∗*P* < 0.001. AA = antimycin A; ATP = adenosine triphosphate; FCCP = carbonyl cyanide-p-trifluoromethoxyphenylhydrazone; ns = not significant; OCR = oxygen consumption rate; SIRT = sirtuins.

**Figure 3:**
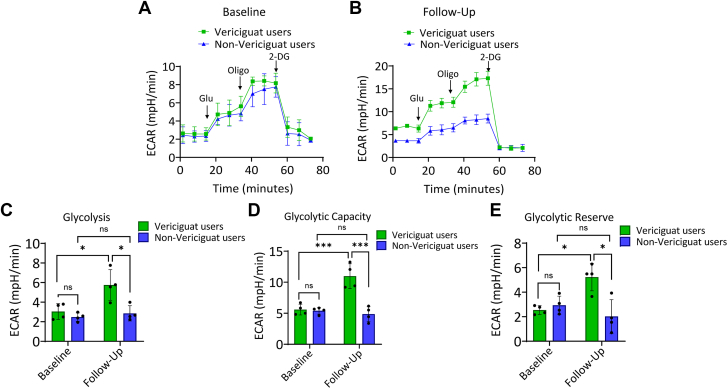
**Vericiguat Enhances Glycolytic Function** In this figure, we evaluated the glycolytic profile of vericiguat users (green color) compared to non-vericiguat users (blue color) at baseline and at the follow-up end. The extracellular acidification rate was measured in real-time using the Seahorse XF Glycolysis Stress Test on peripheral blood mononuclear cells isolated from vericiguat-treated and untreated patients (A, B). Quantification of extracellular acidification rate parameters: (C) glycolysis, (D) glycolytic capacity, and (E) glycolytic reserve. Data are presented as mean ± SD. ∗*P* < 0.05, ∗∗∗*P* < 0.001. ECAR = extracellular acidification rate; ns = not significant.

### Mitochondrial function: cellular bioenergetics analysis and cardiac MIBI

Bioenergetic analysis was performed at baseline and follow-up on PBMCs isolated from the peripheral blood of vericiguat users and non-vericiguat users. At baseline, no significant differences were observed in mitochondrial respiration between the groups (Figures 2A to 2H).

At follow-up end, vericiguat users exhibited a significant improvement in mitochondrial function compared to their own baseline, with higher basal respiration (*P* < 0.01), maximal respiration (*P* < 0.001), mitochondrial ATP production (*P* < 0.01), proton leak (*P* < 0.05), spare respiratory capacity (*P* < 0.001), and nonmitochondrial oxygen consumption (*P* < 0.001) (Figures 2A to 2H).

Then, the mitochondrial SIRT expression was evaluated. Compared to baseline, in vericiguat users at follow-up SIRT3, SIRT4 and SIRT5 protein and mRNA levels were upregulated (*P* < 0.01), suggesting mitochondrial SIRTs as key contributors to the bioenergetic improvement observed during vericiguat treatment (Figures 2I to 2O). Regarding the evaluation of mitochondrial function by MIBI, at follow-up end, we found that the vericiguat users vs nonusers showed the lowest wash out rate (WR) of cardiac MIBI (*P* < 0.05); we did not find significant differences in heart-mediastinum rate (HM) of MIBI (*P* > 0.05) (Figure 1).

### Glycolytic function

At baseline, no significant differences in glycolysis flux were observed between vericiguat users and nonusers (Figures 3A to 3E). Notably, follow-up results showed an increase in ECAR in vericiguat users, with enhanced glycolysis (*P* < 0.05), glycolytic capacity (*P* > 0.001), and glycolytic reserve (*P* < 0.05), suggesting a compensatory increase in glycolysis parallel to the improvement in mitochondrial function (Figures 3A to 3E).

### Anti-inflammatory effect of vericiguat user

**Figure 4:**
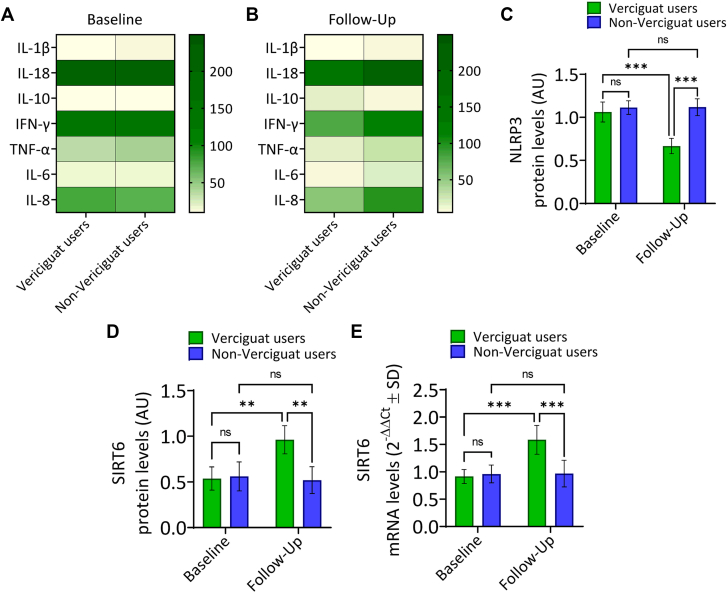
**Vericiguat Reduces Inflammatory Signaling** In this figure, we evaluated the cytokine concentrations in culture supernatants of peripheral blood mononuclear cells from vericiguat-treated patients at baseline (A) and follow-up end (B) by enzyme-linked immunosorbent assays. (C) Quantification of NLRP3 protein expression. (D, E) SIRT6 protein and mRNA levels were evaluated by immunoblotting and quantitative reverse transcription polymerase chain reaction. Data are shown as mean ± SD. ∗∗*P* < 0.01, ∗∗∗*P* < 0.001. IFN = interferon gamma; IL = interleukin; ns = not significant; TNF = tumor necrosis factor; other abbreviation as in Figure 2.

To assess the inflammatory state of PBMCs in response to vericiguat treatment, cytokine secretion was quantified via ELISA at both baseline and follow-up. At baseline, no differences were observed in cytokine release between vericiguat users and nonusers. At follow-up, vericiguat-treated patients exhibited a marked reduction in pro-inflammatory cytokines TNF-α, IL-6, IL-1β, IL-18, and IL-8 compared to baseline levels (*P* < 0.05), suggesting a progressive anti-inflammatory effect associated with vericiguat therapy (Figures 4A and 4B). Conversely, the anti-inflammatory cytokine IL-10 was increased in vericiguat users at follow-up, suggesting a shift toward an immunoregulatory profile (*P* < 0.05) (Figures 4A and 4B).

Moreover, a reduction of the inflammasome-related protein NLRP3 expression was observed at follow-up in vericiguat users (*P* < 0.001), while SIRT6 protein and mRNA levels were upregulated (*P* < 0.01), suggesting a role of SIRT6 as a contributor to the anti-inflammatory effects associated with vericiguat treatment (Figures 4C, 4G, and 4H). Vericiguat treatment improved mitochondrial respiratory capacity and clinical outcomes in CRT non-responder patients. The main findings of the study are summarized in the Central Illustration.

**Central Illustration:**
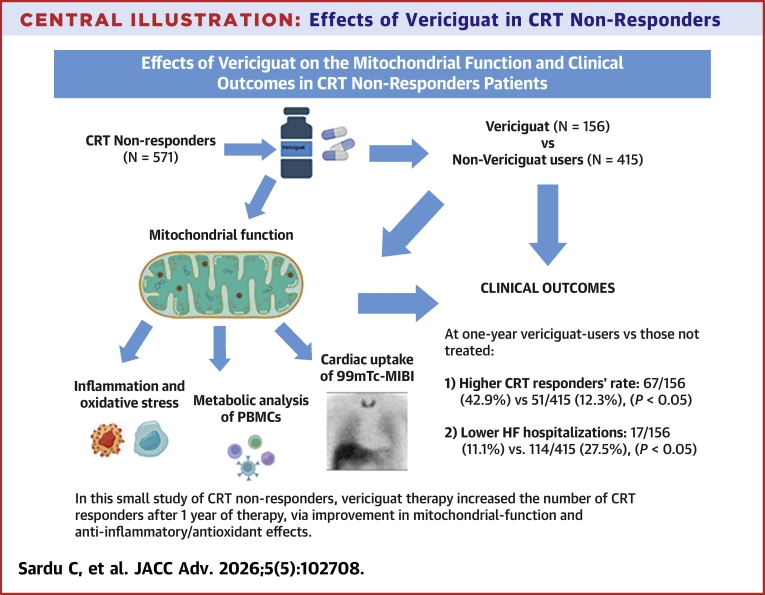
Effects of Vericiguat in CRT Non-Responders In this figure, the representation of cardiac resynchronization therapy nonresponders patients treated with vericiguat therapy (vericiguat-users, n = 156) vs those who did not receive vericiguat (non-vericiguat users, n = 415), and the mitochondrial function assessed through cardiac uptake of technetium-99m methoxyisobutylisonitrile and metabolic analysis of peripheral blood mononuclear cells, and the clinical outcomes (cardiac resynchronization therapy responders and heart failure hospitalizations) at follow-up end. MIBI = methoxyisobutylisonitrile; PBMC = peripheral blood mononuclear cell; other abbreviations as in Figure 1.

## Discussion

Our observational study provides novel, mechanistic insights into the cellular, metabolic, and clinical effects of vericiguat therapy in CRT nonresponders.

At the clinical level, vericiguat significantly increased the rate of CRT responders at 1-year follow-up. As secondary clinical outcomes, vericiguat significantly reduced HF hospitalizations and increased LVEF, 6MWT, and MIBI washout at 1-year follow-up. Intriguingly, vericiguat improves mitochondrial function by enhancing oxidative phosphorylation, increasing ATP production, and reducing inflammatory and oxidative stress pathways, via up-regulation of key mitochondrial SIRT.

Notably, both vericiguat therapy and baseline ATP production were strong predictors of CRT response and HF outcomes, underscoring the central role of mitochondrial dysfunction in CRT nonresponders and highlighting vericiguat as a promising adjunctive therapy for this high-risk population. Specifically, vericiguat therapy and low ATP production predicted a 2.2-fold and 0.3-fold higher likelihood of being CRT responders at 1-year follow-up, respectively, and a 0.3-fold and 4.9-fold change in the risk of HF hospitalization at 1 year, respectively. The sacubitril/valsartan therapy predicted 1.4-fold the CRT responders, and 0.7-fold the HF hospitalizations at 1 year of follow-up. The lowest LVEF values increased about 1.9-fold the risk of HF hospitalizations at 1 year of follow-up.

CRT nonresponders exhibit worsening mitochondrial function, characterized by overinflammation and oxidative stress, reduced mitochondrial membrane potential, and increased MIBI washout.^3^^,^^8^ This could induce LV remodeling, reduce maximal exercise capacity, and worsen NYHA functional class in CRT nonresponders.^3^^,^^8^

Vericiguat therapy significantly reduced the inflammatory cellular and molecular burden (*P* < 0.05) and the NLRP3 expression (*P* < 0.001) in CRT nonresponders. SIRT6 was significantly upregulated, supporting the anti-inflammatory/oxidative and cardioprotective profile,^14^ and induced by vericiguat therapy. Notably, CRT could, per se, decrease myocardial washout and beta-oxidation, leading to a protective state of substrate switching and maintaining mitochondrial redox balance.^8^ These processes decrease myocardial oxygen consumption and the amount of ATP produced per mole of O2 consumed, thereby interfering with the metabolism of fuel pyruvate and the respiratory chain, the site of ATP production.^14^

Targeted metabolomics of peripheral samples, combined with nuclear imaging, may assess substrate utilization and oxidative phosphorylation efficiency in CRT patients.^3^^,^^8^ Indeed, greater mitochondrial dysfunction is associated with reduced fatty acid and acylcarnitine β-oxidation.^8^ In CRT patients, longitudinal metabolite changes significantly correlate with cardiac remodeling and improved clinical outcomes at follow-up.^8^

In our study, vericiguat users vs nonusers showed a significant reduction in the WR of MIBI at follow-up (*P* < 0.05), indicating lower myocardial and altered mediastinal uptake of MIBI.^8^ Notably, decreased ATP synthesis evaluated by metabolic analysis reduced di per se by about 77% of the responsiveness to CRT (CRT responders) and increased the risk of HF hospitalizations by 4.9-fold. At baseline, the study cohorts exhibited comparable metabolic profiles. Subsequently, vericiguat users exhibited a significant increase in mitochondrial function compared to baseline, specifically in basal respiration (*P* < 0.05), maximal respiration (*P* < 0.01), mitochondrial ATP production (*P* < 0.01), and proton leak (*P* < 0.05). Indeed, vericiguat preserves mitochondrial quality in the heart^15^ by inducing myocyte NO synthesis, which triggers PKG activity and improves cardiac function in rats.^16^ Higher cGMP levels mediate vasodilation via antifibrotic/inflammatory effects.^17^^,^^18^ We confirmed these effects, with a significant increase in OCR in vericiguat users compared to nonusers at the follow-up end (*P* < 0.05), indicating enhanced mitochondrial efficiency.

Intriguingly, at follow-up, we observed increased ECAR parameters in vericiguat users, suggesting that vericiguat could promote glycolysis and improve overall cellular bioenergetic capacity in CRT nonresponders. This observation is further supported by the increase in basal glycolysis and higher total ATP production (*P* < 0.05) in vericiguat users vs nonusers at follow-up. Moreover, vericiguat users at follow-up showed mitochondrial SIRT SIRT3, SIRT4, and SIRT5 up-regulation, key regulators of mitochondrial integrity, oxidative metabolism, and cellular response to oxidative stress and inflammation.^19^ These findings suggest that vericiguat may exert pleiotropic effects through activation of mitochondrial SIRT pathways, thus enhancing bioenergetic efficiency and stress adaptation in CRT cohorts.^20^

At the clinical level, vericiguat therapy could increase the CRT responder outcome by 2.2-fold and reduce hospitalizations for HF worsening by approximately 70%. Indeed, the vericiguat is a direct stimulator of NO-sGC-cGMP pathways that regulates the cardiovascular system by catalyzing cGMP synthesis in response to NO and prevents myocardial and vascular dysfunction associated with decreased sGC activity in the HF model.^19^ These effects reduce cardiac fibrosis and remodeling, resulting in optimal LV contractility.^19^ Similarly, sacubitril/valsartan increased the CRT responders by about 1.4-fold and reduced the risk of HF worsening hospitalizations by about 30% at 1 year of follow-up. This confirms the sacubitril/valsartan ameliorative molecular and cellular effects via antiremodeling properties, and best clinical outcomes in CRT nonresponders via epigenetic modulation.^2^ In this setting, the reduced LVEF significantly increased the risk of HF hospitalizations at the follow-up end. LVEF, assessed by echocardiography, is a key indicator of cardiac pump function.^4^ In CRT nonresponders, severely reduced LVEF reflects adverse remodeling and advanced HF and is associated with worse prognosis and higher hospitalization risk.^2^^,^^4^

### Study limitations

The study evidences several limitations. First, we did not collect coronary sinus serum samples because of the risk of LV lead dislodgment in CRT nonresponders. Second, myocardial tissue was unavailable in the study cohorts, and bioenergetic and molecular analyses were performed on PBMCs. Although PBMCs are widely used to investigate systemic metabolic and inflammatory adaptations in cardiovascular disease, they may not fully reflect the specific mitochondrial and inflammatory changes that occur within cardiac cells. However, PBMCs provide insight into systemic oxidative, inflammatory, and metabolic pathways that may complement myocardial findings. Furthermore, mitochondrial biogenesis has not been directly evaluated, and its potential contribution to the observed improvements in mitochondrial function cannot be excluded. Data from this study primarily support an increase in functional efficiency rather than biogenesis, but this aspect requires further investigation. Third, we did not use experimental models to investigate calcium channel expression and did not test drugs to block or enhance mitochondrial function, such as mitoK-ATP modulators and calcium-channel blockers, such as nicardipine.

Again, we did not investigate metabolite concentrations (via peripheral venous blood) at baseline and follow-up, which are implied in mitochondrial function and cardiac metabolism.^7^ Thus, we did not report differences in acylcarnitines, amino acids (free fatty acids, acylcarnitine ratio, and glutamate), or in the acylcarnitine ratio between baseline and follow-up. Furthermore, this could limit the study's results because there is no conclusive data on mitochondrial fatty acid oxidation, long-chain fatty acid oxidation, and the CRT-induced changes in metabolites of ketone metabolism and glycolysis, ketone bodies, and lactate production.^7^ On the other hand, these results were well-evidenced and discussed in CRT cohorts.^7^

Furthermore, we do not have conclusive data on mitochondrial fatty acid oxidation, long-chain fatty acid oxidation, and the CRT-induced changes in metabolites of ketone metabolism and glycolysis, ketone bodies, and lactate production.^7^ On the other hand, these results were well-evidenced and discussed in CRT cohorts.^7^

Again, in the current study, we defined time zero as the initiation of vericiguat therapy for users and baseline evaluation for nonusers. This may introduce immortal time bias, potentially excluding more severe cases among nonusers who might have been eligible for vericiguat therapy. In this context, the absence of propensity score matching may have resulted in residual confounding. Finally, the short follow-up duration and the size of the study population could affect the clinical endpoints investigated and the study results.

## Conclusions

In CRT nonresponders, vericiguat therapy is associated with improved mitochondrial function, more efficient myocardial energy metabolism, and favorable ventricular remodeling. These changes translate into better functional capacity with a higher CRT responders’ rate and fewer HF hospitalizations at 1 year. These findings suggest that targeting mitochondrial dysfunction may offer a clinically meaningful therapeutic strategy to improve outcomes in patients who fail to respond to CRT.

Perspectives **COMPETENCY IN MEDICAL KNOWLEDGE:** In CRT nonresponders, the addition of vericiguat improves systemic mitochondrial efficiency and reduces inflammatory/oxidative stress. These ameliorative effects were associated with reduced myocardial MIBI washout at 12 months, consistent with improved in vivo mitochondrial function.

**COMPETENCY IN PATIENT CARE:** In an observational cohort of CRT nonresponders (n 571), patients treated with vericiguat showed higher conversion to CRT responders (42.9% vs 12.3%), reduced HF hospitalizations (11.1% vs 27.5%), functional improvement (NYHA class, 6MWT distance), increased LVEF, and favorable LV remodeling. These results suggest that selection of patients with energetic dysfunction (low PBMC and ATP levels) may identify a subgroup deriving greater benefit from adjunctive vericiguat therapy.

**TRANSLATIONAL OUTLOOK:** The efficacy of vericiguat appears to be mediated by restoration of the NO-sGC-cGMP pathway, impacting mitochondrial metabolism and anti-inflammatory pathways. These findings support the use of PBMC bioenergetics and SIRT3 activity as potential surrogate biomarkers of myocardial mitochondrial status, to be validated in prospective randomized studies. In the future, a phenotype-guided approach integrating systemic energetic profiling with clinical and echocardiographic criteria may enable personalized therapy with vericiguat in CRT nonresponders.

## Funding support and author disclosures

The authors have reported that they have no relationships relevant to the contents of this paper to disclose.
