# Supplementary material for: Effects of Vericiguat on the Mitochondrial Function and Clinical Outcomes in CRT Nonresponders Patients
Source: JACC Adv. 2026 Apr 4;5(5):102708. doi: 10.1016/j.jacadv.2026.102708 (PMC13089008; doi:10.1016/j.jacadv.2026.102708)
Supplement: Supplemental Material [file mmc1.docx]

**Supplementary files.**

**Effects of vericiguat on the mitochondrial function and clinical outcomes in CRT non-responders patients**

Celestino Sardu, MD, MSc, PhD1,2*; Nunzia D’Onofrio, MD3,4; Carlo Fumagalli, MD1,2; Maria Luisa Balestrieri, MD3,4; Alessandro Landolfi, MD2; Carmine La Marca, MD5; Valerio Giordano, MD6; Isabella Donisi, MD3; Pietro Rambaldi, MD7; Gianluca Gatta, MD7; Salvatore Cappabianca, MD7; Ferdinando Carlo Sasso, MD1; Michelangela Barbieri, MD1; Giuseppe Paolisso, MD1,4,8; Raffaele Marfella MD, PhD1,4

1. Department of Advanced Medical and Surgical Sciences, University of Campania Luigi Vanvitelli, Naples, Italy; 2. Department of Neuro-Cardiovascular Sciences, IRCCS Neuromed, Pozzilli, Italy; 3. Department of Experimental Medicine, University of Campania Luigi Vanvitelli, Naples, Italy; 4. Research Center for Environmental Pollution and Cardiovascular Diseases, University of Campania Luigi Vanvitelli, Naples, Italy; 5. Department of Cardiovascular Sciences, Santa Maria della Pietà Hospital, Nola, Naples, Italy; 6. Department of Cardiovascular Sciences, San Luca Hospital Hospital, Vallo della Lucania Italy; 7. Department of Precision Medicine, University of Campania Luigi Vanvitelli, Naples, Italy; 8. School of Medicine, “Saint Camillus University”, Rome, Italy.

**Running title**: mitochondrial dysfunction and vericiguat.

***Corresponding author:**

Celestino Sardu, MD, MSc, PhD Piazza Miraglia, 2; 80138, Naples. Italy; Telephone: +39 0815665110; fax: +39 0815095303; email: drsarducele@gmail.com

**Word count:** 1252.

**METHODS**

**Technetium 99m sestamibi** **(MIBI) cardiac scintigraphy**

We performed, at enrollment and at follow-up end, the Technetium 99m sestamibi (MIBI) imaging study. We injected the MIBI (600 MBq; Daiichi Radioisotope Laboratories Ltd, Tokyo, Japan) via peripheral vein access, and we obtained the myocardial image with a standard-field gamma camera equipped with a low-energy, general-purpose collimator (Ecam Dual Head, Siemens, Germany), using a 20% window centered at 140 keV Early and delayed images were obtained 1 and 3 hours after the injection, respectively. We used images obtained 1 hour after the injection as the early images, because accumulation of MIBI in the myocardium is not significantly altered at 30 to 60 minutes after the injection of the tracer, even though the liver accumulation decreases with time; we used a subsequent 2-hour interval to obtain delayed images, as previously reported (**1**).

According to the weight of the patients, we subministered 12 mCi (patients weighing less than 75 kg) and/or received 15 mCi ^99m^TC-sestamibi (patients weighing more than 75 kg). Early images were acquired 60 minutes and late images 240 minutes after tracer injection on a Siemens T6 SPECT/CT. We used planar images to calculate the myocardial wash-out rate (WR) by comparing early and late heart-to-mediastinum (H/M) region of interest counts (**1**). We traced manually the regions of interest, over the heart and mediastinum, with the same template used for the early and late images. Nuclear analysis was performed by experienced technicians blinded to the patients clinical status and study protocol. The WR was calculated by subtracting late H/M count corrected for tracer decay from the early H/M count and indexing for early H/M count (**1**).

**Image Analysis**

We acquired the MIBI planar images (512 pixels) of the anterior view of the thorax, obtaining the single photon emission computed tomography (SPECT) data over 180° in 60 steps, each of which was 30 seconds. We acquired eight frames (64 pixels) per R-R interval of the electrocardiogram (**1**) . We processed the non-gated SPECT data of MIBI by filtered back projection (eighth-order Butterworth filter with a cutoff frequency of 0.5 cycles per pixel, and tomograms with a slice thickness of 5.73 mm). Then, we reconstructed the data in transverse-axial, short-axial, and vertical long axial tomograms, to calculate the left ventricular end-diastolic volume (LVEDV) index, left ventricular end-systolic volume (LVESV) index, and left ventricular ejection fraction (LVEF) data with quantitative electrocardiography-gated SPECT programs (**1**).

We quantified the uptake of the tracer with processing equipment (Siemens, Germany), and then calculated the heart-to-mediastinum (H/M) ratio and the WR for MIBI; left ventricular activity and mediastinum activity were measured by use of manually drawn regions of interest covering the entire left ventricular myocardium and the upper mediastinum area, respectively, and the mean heart counts per pixel were calculated in each region (**1**). The H/M ratio was calculated from the heart and mediastinum planar images of the anterior thorax as follows: H/M ratio = [H]delayed /[M]delayed, where [H] and [M] are the mean counts per pixel in the left ventricle and upper mediastinum, respectively. The WR was defined as the percentage change in activity from early to delayed images as follows: WR = ([([H] - [M])early - ([H] - [M])delayed]/([H] - [M])early) - 100 (%), (**1**)

**Laboratory analysis**

From peripheral venous samples obtained from all patients after an overnight fast, we measured plasma glucose, serum lipids, BNP, and NT-proBNP using enzymatic assays. Fasting blood (at least 12 h from the last meal) was collected for biochemical assays at baseline and at the follow-up visit. Blood samples were collected in an ice-cooled blood collection system and immediately centrifuged at 2500 rpm for 10 min in a refrigerated centrifuge. Samples were stored at − 80 ◦C. Inflammatory and oxidative stress markers were measured in the study cohorts at baseline and follow-up in duplicate using a highly sensitive quantitative sandwich enzyme-linked immunosorbent assay (ELISA, Quantikine HS; R&D Systems, Minneapolis, MN). Inflammatory molecular and cellular markers included circulating serum levels of pro-inflammatory cytokines (tumor necrosis factor-α, TNF-α, interleukin-6, IL6), inflammatory markers (C-reactive protein, CRP), leukocyte and neutrophil counts. Oxidative stress was assessed by serum Nitrotyrosine levels by ELISA after an overnight fast, at breakfast time, and before sensor insertion.

**PBMCs Isolation**

Peripheral blood was collected from vericiguat treated and from untreated patients, using heparinized tubes. For each sample, 10 ml of blood was drawn, and was processed within 2 hours of collection to ensure cell viability. Peripheral blood mononuclear cells (PBMCs) were isolated using density gradient centrifugation with Histopaque-1077 (10771, Sigma, St Louis, MO, USA). The collected blood was diluted 1:1 with phosphate-buffered saline (PBS) and carefully layered over Histopaque-1077 at a 1:2 ratio. The tubes were then centrifuged at 400 xg for 30 minutes at 4°C with no brake, allowing the cells to separate according to their density. After centrifugation, lymphocyte layer was carefully collected and transferred to a 15 mL Falcon tube. The cells were washed twice with PBS by centrifuging at 300 g for 10 minutes at 4°C. After washing, cells were resuspended in RPMI 1640 medium (L0500, Aurogene) supplemented with 10% fetal bovine serum (FBS, 10270-106, Gibco, Life Technologies, Carlsbad, CA, USA) and incubated at 37°C in a humidified atmosphere with 5% CO₂. PBMC isolation was performed to enable measurement of mitochondrial bioenergetics, including oxygen consumption rate (OCR), adenosine triphosphate (ATP) production, proton leak, glycolytic flux, and cytokine secretion profiles (**10**).

**Metabolic Analysis**

Cellular bioenergetics were assessed using the Seahorse XF HS Mini Analyzer (Agilent Technologies, Santa Clara, CA, USA). Mitochondrial respiration was assessed using the Seahorse Cell MitoStress Test Kit (103010-100, Agilent). The sensor cartridge was hydrated in XF calibrant (103059-000, Agilent) at 37°C in a non-CO2 incubator. After isolation, cells were seeded in Seahorse assay microplates at the density of 2 × 104 cells per well in Seahorse XF DMEM medium supplemented with 10 mM glucose, 2 mM L-glutamine and 1 mM pyruvate (103575-100, 103577-100, 103579-100, 103578-100, Agilent) and incubated at 37°C in a non-CO₂ incubator for 1 h before analysis. During the assay, sequential injections of 1.5 µM oligomycin, 1 µM FCCP, and 0.5 µM rotenone + antimycin A were administered to evaluate key mitochondrial respiration parameters. Glycolysis was assessed using the Seahorse XF Glycolysis Stress Test Kit (103020-100, Agilent). Sequential additions of 10 mM glucose, 1 µM oligomycin, and 50 mM 2-deoxy-D-glucose (2-DG) were performed to determine glycolytic flux. Data were normalized to the protein content of each well.

**Glycolytic Function Assay**

Glycolytic flux in PBMCs was assessed using extracellular acidification rate (ECAR) measurements obtained with the Seahorse XF HS Mini Analyzer (Agilent Technologies, Santa Clara, CA, USA). After PBMCs isolation, cells were plated in specialized microplates and sequentially exposed to glucose, oligomycin, and 2-deoxyglucose to quantify key glycolytic parameters, including basal glycolysis, glycolytic capacity, and glycolytic reserve. The Extracellular acidification rate (ECAR) was measured in real-time using the Seahorse XF Glycolysis Stress Test on PBMCs isolated from vericiguat-treated and untreated patients. ECAR values were normalized to cell numbers and analyzed. These measurements allowed evaluation of glycolytic adaptations at baseline and during follow-up in vericiguat users and non-users. However, we quantified ECAR parameters, including glycolysis, glycolytic capacity, and glycolytic reserve.

**SIRT3 Enzymatic Activity Assay**

Sirtuin 3 (SIRT3) enzymatic activity was assessed using the Fluor de Lys® SIRT3 Fluorometric Drug Discovery Assay Kit (BML-AK557, Enzo Life Sciences). Equal amounts of total protein were incubated with the acetylated fluorogenic substrate in the presence of NAD⁺ for 60 minutes. After incubation, the developing solution was added, and the reaction was allowed to proceed for 15 minutes at room temperature. Fluorescence intensity was measured at an excitation wavelength of 360 nm and an emission wavelength of 460 nm using a fluorescence microplate reader (Tecan Infinite 2000 Multiplate reader, Tecan, Männedorf, Switzerland). Fluorescence values were recorded as arbitrary fluorescence units (AFU) and SIRT3 enzymatic activity in each sample was expressed as a percentage relative to the mean activity observed in non-vericiguat users, which was set as 100%.

**Immunoblotting analysis**

Protein expression was analyzed by Western blotting. The cells were lysed in RIPA buffer supplemented with protease and phosphatase inhibitor cocktail, and protein concentrations were detected using the Bio-Rad Protein Assay kit (Bio-Rad). Equal amounts of protein were denatured in Laemmli buffer at 95°C for 10 minutes and separated by sodium dodecyl sulfate–polyacrylamide gel electrophoresis (SDS-PAGE). Proteins were transferred to a nitrocellulose membrane and blocked for 1 hour at room temperature. Membranes were incubated overnight at 4°C with anti-sirtuin 3 (SIRT3, 1:500, #2627, Cell Signaling Technology), anti-sirtuin 4 (SIRT4, 1:1000, E-AB-16821, Elabscience), anti-sirtuin 5 (SIRT5, 1:1000, E-AB-15844, Elabscience), anti-sirtuin 6 (SIRT6, 1:1000, ab62738, Abcam) and NLR Family Pyrin Domain Containing 3 (NLRP3, 1:1000, ab4207, Abcam) primary antibodies diluted in blocking buffer. After washing with TBS-T, membranes were incubated with horseradish peroxidase (HRP)-conjugated secondary antibodies for 1 hour at room temperature. Immunoreactive bands were visualized using enhanced chemiluminescence (ECL) detection reagents, and imaged using a ChemiDoc Imaging System (Bio-Rad). Band intensities were quantified using ImageJ software and target protein levels were normalized to the corresponding loading control. The immunoblotting analysis was used to quantify the expression of mitochondrial sirtuins (SIRT3, SIRT4, SIRT5), SIRT6, and the inflammasome protein NLRP3, which were evaluated as mechanistic markers of mitochondrial function, oxidative stress, and inflammatory signaling (**12,13**).

**ELISA assays**

After isolation, cells were seeded at a density of 1 × 10⁶ cells per well in 24-well culture plates and maintained at 37°C in a humidified incubator with 5% CO₂. Supernatants were collected after 24 hours of incubation and centrifuged at 300 g for 5 minutes to remove any residual cells or debris. The concentrations of secreted cytokines, including Interferon gamma (IFN-γ; ab174443, Abcam), TNF-α (ab181421, Abcam), IL-6 (ELK1156, ELK Biotechnology), IL-18 (ab215539, Abcam), IL-1β (ab214025, Abcam) and IL-10 (ab174443), IL-8 (ab46032, Abcam) were measured using enzyme-linked immunosorbent assay (ELISA) kits specific for human targets. The supernatant was incubated for 1 hour in precoated plates with a specific antibody cocktail. Absorbance was read at 450 nm using a microplate reader (model 680, Bio-Rad). Cytokine concentrations and NLRP3 levels were calculated by plotting absorbance values against each standard curve.

**Statistical analysis**

Continuous variables were expressed as means and standard deviations and tested by a two-tailed Student T-test. We compared categorical variables using the Chi-square or Fisher’s exact test, as appropriate. To evaluate longitudinal changes in the study variables, echocardiographic parameters, biomarkers of inflammation, medications, bioenergetic profile of PBMCs, mitochondrial respiration, sirtuins expression, and cytokine secretion and inflammasome-related signaling in vericiguat-users vs non-users, the data collected at baseline and at one-year follow-up were analyzed using linear mixed models (LMM) for repeated measures, with time considered as a within-subject factor. LMMs analysed the longitudinal changes within and between groups, with time as a within-subject factor and treatment group as a between-subject factor. The group × time interaction was tested to evaluate whether trajectories of change differed between groups.

In all LMM analyses, treatment group (vericiguat users vs non-users) was modeled as a fixed effect, while time was modeled as a random effect to account for within-subject correlation across repeated measurements. We included the intraclass correlation coefficients (ICCs) for the main models, along with a description of the model assumptions and diagnostic checks (including assessment of normality of residuals and homoscedasticity). LMM analyses were adjusted for age, sex, baseline NYHA functional class, baseline LVEF, ischemic etiology of heart failure, renal function (eGFR), and background guideline-directed medical therapy (including β-blockers, ACE inhibitors/ARBs or sacubitril/valsartan, and mineralocorticoid receptor antagonists). **Supplementary table 2**.

As a sensitivity analysis, a Poisson regression model was performed to evaluate CRT responders and HF hospitalization rates at 12 months.

**RESULTS**

We reported the results of data comparison of follow-up end vs. baseline condition in each cohort

of study: vericiguat-users and non-vericiguat users.

**-Vericiguat-users:** at 1 year of follow-up vs. baseline condition, the vericiguat-users showed a higher rate of patients in I and II New York Heart Association (NYHA) class, and a lower rate of patients in III and IV NYHA class, with a significant increase of six-minutes walking test (6MWT), (p<0.05). **Supplementary table 1**. By analysis of echocardiographic data, we noted a significant reduction in cardiac dimension and stage of mitral valve insufficiency, and a significant increase of left ventricle ejection fraction (LVEF), (p<0.05). **Supplementary table 1.**

At follow-up end these patients evidenced significant reduction of inflammatory/oxidative stress markers, B type natriuretic peptide (BNP) and N-terminal pro-BNP (NT-proBNP), (p<0.05). **Supplementary table 1.**

**-Non-vericiguat users:** comparing the follow-up end vs. baseline condition, the non-vericiguat

showed a worsening of NYHA class, and of mitral valve insufficiency (p<0.05). **Supplementary**

**table 1**. These patients showed a significant reduction of inflammatory/oxidative stress markers and

of NT-proBNP, while the BNP values significantly increased (p<0.05). **Supplementary table 1**.

The non-vericiguat users showed a significant increase of sacubitril/valsartan, ivabradine and

sodium glucose transporter 2 inhibitors (SGLT2i) treatment at follow-up end (p<0.05).

**Supplementary table 1**. In supplementary table 2, we reported the results of the LMM approach.

Model diagnostics confirmed the adequacy of the analysis, showing moderate between-subject

variability (ICC = 0.37) and no violations of assumptions regarding normality, homoscedasticity,

linearity, or influential outliers. In supplementary table 3 we reported the results obtained by

Poisson regression analysis to evaluate CRT responders (A) and HF hospitalization (B) rates at 12

months. The results were consistent with those obtained from the Cox model, showing similar

effect estimates for vericiguat and other covariates.

**DISCUSSION**

Vericiguat could modulate the mitochondrial biogenesis and function in CRTd non-responders via anti-inflammatory/oxidative properties. Vericiguat could significantly reduce BNP and NT-proBNP peptides serum levels and induce cardiac remodelling with the increase of LVEF at follow-up end in CRT non-responders. These effects could ameliorate the clinical status in CRT non-responders cohorts. In specular manner, the Non-vericiguat users showed the worsening of NYHA class and the significant increase of BNP levels, and the rate of patients under sacubitril/valsartan and SGLT2i therapy.

Vericiguat therapy could ameliorate the myocardial mitochondrial function, by inducing the normalization of global substrate use, and the improvement in exercise capacity, and leading to the reverse LV remodelling in the CRT non-responders. The mitochondrial dysfunction and the over inflammatory/oxidative stress in CRT non-responders could cause the worse prognosis (**3**). The mitochondria are unique organelles, that could lead to an increase of adenylyl cyclase activity by the import of anions through the outer membrane and voltage dependent anion channels (**4**). The soluble adenylyl cyclase is regulated by intramitochondrial bicarbonate and calcium and exerts short-term regulation of cytochrome c-oxidase via phosphorylation of its protein subunits (**5**). However, the cardiac mitochondrial dysfunction causes accumulation of metabolic products, oxidative damage, impaired mitochondrial function and cell death, leading to structural remodeling via hypertrophy and myocardial fibrosis in animal models (**4-6**). In a vicious circle, this increases the production of inflammatory/oxidative molecules (**7**), induces a mitochondrial complex defect and DNA damage (**8**), and causes apoptosis in cultured cardiomyocytes (**9**). The inflammatory cytokines contribute di per se to the development of the systemic bioenergetic failure, while the “in vivo” tumor necrosis factor alpha (TNFα) inhibition normalizes mitochondrial function and oxidative stress and decreases apoptosis in this experimental HF-model (**10**). Notably, the mitochondrial ATP synthase function is attenuated in HF, and restored (at least in part) by CRT ameliorative effects (**11**). Indeed, CRT affects the mitochondrial sub-proteome by altering specific proteins that control the cellular redox state and oxidative phosphorylation pathways, as manifested by changes in both protein quantity and post-translational modifications within the mitochondria (**11**). These effects are reduced to total loss in CRT non-responders (**12**). In CRT non-responders the over-inflammation/oxidative stress and the mitochondrial dysfunction condition the cardiac metabolism, the clinical status and lead to the worse prognosis (**12**). Indeed, the healthy myocytes synthetize the nitric oxide (NO), which stimulates the production of cyclic guanosine monophosphate (cGMP), triggering the activity of protein-kinase G (PKG), which modulates several targets involved in myocyte contraction, and thereby regulating cardiac function in rats (**13**). In addition, the cGMP mediates the vasodilation, exerting anti-fibrotic, and anti-inflammatory effects (**14**). In this setting, the vericiguat restores the impaired NO–soluble guanylate cyclase (sGC)– cGMP pathways in the cardiac cells (**2, 5**). To date, according to our study results, we could consider the vericiguat as a c-GMP stimulator and activator of mitochondrial function in CRT non-responders. This effect, reducing the inflammatory/oxidative stress and favouring the mitochondrial bioenergetic, could cause LV reverse remodelling with the increase of cardiac pump. These molecular, cellular and mechanical cardiac effects could result in the amelioration of clinical status and prognosis in CRT non-responders patients.

**REFERENCES**

1. Sugiura T, Takase H, Toriyama T, Goto T, Ueda R, Dohi Y. Usefulness of Tc-99m methoxyisobutylisonitrile scintigraphy for evaluating congestive heart failure. J Nucl Cardiol. 2006 Jan-Feb;13(1):64-8. doi: 10.1016/j.nuclcard.2005.10.003.

2. 7. Heidenreich PA, Bozkurt B, Aguilar D, et al. 2022 AHA/ACC/HFSA Guideline for the Management of Heart Failure: A Report of the American College of Cardiology/American Heart Association Joint Committee on Clinical Practice Guidelines. Circulation. 2022 May 3;145(18):e895-e1032. doi: 10.1161/CIR.0000000000001063.

3. Martens P, Dupont M, Vermeersch P, Dauw J, Nijst P, Bito V, Mesotten L, Penders J, Janssens S, Tang WHW, Mullens W. Impact of Cardiac Resynchronization Therapy on Global and Cardiac Metabolism and Cardiac Mitochondrial Function. J Card Fail. 2021 Jun;27(6):706-715. doi: 10.1016/j.cardfail.2021.02.008.

4. Werbner B, Tavakoli-Rouzbehani OM, Fatahian AN, Boudina S. The dynamic interplay between cardiac mitochondrial health and myocardial structural remodeling in metabolic heart disease, aging, and heart failure. J Cardiovasc Aging. 2023 Jan;3(1):9. doi: 10.20517/jca.2022.42.

5. Jungtanasomboon P, Nussaro S, Winwan H, Suebthawinkul P, Boonpala P, Dong VNK, Saengklub N, Kumphune S, Panyasing Y, Kijtawornrat A. Vericiguat preserved cardiac function and mitochondrial quality in a rat model of mitral regurgitation. Life Sci. 2023 Sep 1;328:121929. doi: 10.1016/j.lfs.2023.121929.

6. Zhu W, Ben Y, Shen Y, Liu W. Vericiguat protects against cardiac damage in a pig model of ischemia/reperfusion. PLoS One. 2023 Dec 22;18(12):e0295566. doi: 10.1371/journal.pone.0295566.

7. Ozcan C, Bienengraeber M, Hodgson DM, Mann DL, Terzic A. Mitochondrial tolerance to stress impaired in failing heart. J Mol Cell Cardiol. 2003 Sep;35(9):1161-6. doi: 10.1016/s0022-2828(03)00204-9.

8. Suematsu N, Tsutsui H, Wen J, Kang D, Ikeuchi M, Ide T, Hayashidani S, Shiomi T, Kubota T, Hamasaki N, Takeshita A. Oxidative stress mediates tumor necrosis factor-alpha-induced mitochondrial DNA damage and dysfunction in cardiac myocytes. Circulation. 2003 Mar 18;107(10):1418-23. doi: 10.1161/01.cir.0000055318.09997.

9. Song W, Lu X, Feng Q. Tumor necrosis factor-alpha induces apoptosis via inducible nitric oxide synthase in neonatal mouse cardiomyocytes. Cardiovasc Res. 2000 Feb;45(3):595-602. doi: 10.1016/s0008-6363(99)00395-8.

10. Moe GW, Marin-Garcia J, Konig A, Goldenthal M, Lu X, Feng Q. In vivo TNF-alpha inhibition ameliorates cardiac mitochondrial dysfunction, oxidative stress, and apoptosis in experimental heart failure. Am J Physiol Heart Circ Physiol. 2004 Oct;287(4):H1813-20. doi: 10.1152/ajpheart.00036.2004.

11. Agnetti G, Kaludercic N, Kane LA, Elliott ST, Guo Y, Chakir K, Samantapudi D, Paolocci N, Tomaselli GF, Kass DA, Van Eyk JE. Modulation of mitochondrial proteome and improved mitochondrial function by biventricular pacing of dyssynchronous failing hearts. *Circ Cardiovasc Genet*. 2010;*3*:78–87.

12. Martens P, Dupont M, Vermeersch P, Dauw J, Nijst P, Bito V, Mesotten L, Penders J, Janssens S, Tang WHW, Mullens W. Impact of Cardiac Resynchronization Therapy on Global and Cardiac Metabolism and Cardiac Mitochondrial Function. J Card Fail. 2021 Jun;27(6):706-715. doi: 10.1016/j.cardfail.2021.02.008.

13. J. Layland, J.M. Li, A.M. Shah. Role of cyclic GMP-dependent protein kinase in the contractile response to exogenous nitric oxide in rat cardiac myocytes. J. Physiol., 540 (Pt 2) (2002), pp. 457-467.

14. M. Gheorghiade, C.N. Marti, H.N. Sabbah, L. Roessig, S.J. Greene, M. Bohm, et al. Soluble guanylate cyclase: a potential therapeutic target for heart failure. Heart Fail. Rev., 18 (2) (2013), pp. 123-134.

**Table and figures legend**

**Supplementary table 1.** In this table the clinical characteristics of vericiguat-users and non-vericiguat users, as the 1 year of follow-up vs. baseline data (in each cohort of patients).

A.U: arbitrary units; BMI: body mass index; COPD: chronic obstructive pulmonary disease; IDCM: ischemic dilated cardiomyopathy; NYHA: New York Heart Association; 6MWT: 6 minutes walking test; LVEF: left ventricle ejection fraction; LVEDd: left ventricle end-diastolic diameter; LVESd: left ventricle end-systolic diameter; LVEDv: left ventricle end-diastolic volume; LVESv: left ventricle end-systolic volume; LNR: Lymphocytes/Neutrophils ratio; BNP: B-type natriuretic peptide; NT-proBNP: N terminal pro-B type natriuretic peptide; CRP: C reactive protein; ACE: Angiotensin converting enzyme; ARS: Angiotensin receptors blockers; NOAC: new oral anticoagulants; SGLT2i: sodium glucose transporter 2 inhibitors; GLP-1: Glucagon-like peptide-1; DPP-4: Dipeptidyl peptidase 4; *: statistical significant (p<0.05).

**Supplementary table 2.** In this table the linear mixed model (LMM) approach. The treatment group (vericiguat users vs non-users) was modeled as a fixed effect, while time was modeled as a random effect to account for within-subject correlation across repeated measurements. We included the intraclass correlation coefficients (ICCs) for the main models, along with a description of the model assumptions and diagnostic checks (including assessment of normality of residuals and homoscedasticity). *: statistical significant (p<0.05).

**Supplementary table 3.** In this table the Poisson regression analysis as a sensitivity analysis to evaluate Cardiac resynchronization therapy (CRT) responders (A) and Heart failure hospitalization (B) rates at 12 months. ATP: adenosine triphosphate; LVEF: left ventricle ejection fraction; *: statistical significant (p<0.05).

**Supplementary Figure S1.** Ponceau staining and full-length Western blot images at baseline. Representative Ponceau staining (left panels) and corresponding full-length Western blots (right panels) of mitochondrial sirtuins (SIRT3, SIRT4, and SIRT5) in PBMCs from vericiguat users and non vericiguat users at baseline. Actin was used as loading control. M, molecular weight markers; lane 1, Vericiguat users; lane 2, Non-Vericiguat users.

**Supplementary Figure S2**. Full-length Western blots and Ponceau staining at follow-up. Representative Ponceau staining (left panels) and corresponding full-length Western blots (right panels) of mitochondrial sirtuins (SIRT3, SIRT4, and SIRT5), evaluated in PBMCs from vericiguat-treated patients at the end of follow-up. Actin was used as loading control. M, molecular weight markers; lane 1, Vericiguat users; lane 2, Non-vericiguat users.

**Supplementary Figure S3**. Ponceau staining and full-length Western blot images of NLRP3 and SIRT6 at baseline and follow-up. Representative Ponceau staining (left panels) and corresponding full-length Western blots (right panels) of NLRP3 and SIRT6 in PBMCs from vericiguat users and non-users at baseline and at the end of follow-up. α-Tubulin was used as loading control. M, molecular weight markers; lane 1, Vericiguat users; lane 2, Non-vericiguat users.

**Supplementary table 1.** Clinical characteristics of vericiguat-users and non-vericiguat users, as the 1 year of follow-up vs. baseline data.

| **STUDY VARIABLES** | **Vericiguat-users**  **n 156**  **(baseline)** | **Vericiguat-users**  **n 156**  **(follow-up end)** | **p value** | **Non-Vericiguat users**  **n 415**  **(baseline)** | **Non-Vericiguat users**  **n 415**  **(follow-up end)** | **p value** |
| --- | --- | --- | --- | --- | --- | --- |
| Age, years | 71.4±4.6 | / | / | 71.5±6.4 | / | / |
| Male, n (%) | 96 (61.5) | / | / | 289 (69.6) | / | / |
| BMI >30 Kg/m2, n (%) | 12 (7.7) | 10 (6.4) | 0.782 | 29 (7.0) | 36 (8.7) | 0.366 |
| Smokers, n (%) | 85 (54.5) | 92 (59.0) | 0.424 | 237 (57.1) | 250 (60.2) | 0.574 |
| Hypertension, n (%) | 110 (70.5) | 114 (73.1) | 0.526 | 311 (74.9) | 331 (80) | 0.415 |
| Diabetes mellitus, n (%) | 109 (69.9) | 113 (72.4) | 0.435 | 270 (65.1) | 278 (67) | 0.892 |
| Dyslipidemia, n (%) | 76 (48.7) | 69 (44.2) | 0.256 | 209 (50.4) | 190 (45.8) | 0.455 |
| COPD, n (%) | 41 (26.3) | 43 (27.6) | 0.799 | 115 (27.7) | 120 (28.9) | 0.415 |
| IDCM, n (%) | 93 (59.6) | / | / | 235 (56.6) | / | / |
| I NYHA class, n (%) | / | 5 (3.2) | 0.001* | / | / | 0.005* |
| II NYHA class, n (%) | 65 (41.7) | 85 (54.5) |  | 166 (40.0) | 111 (26.7) |  |
| III NYHA class, n (%) | 73 (46.8) | 59 (37.8) |  | 206 (49.6) | 253 (61.0) |  |
| IV NYHA class, n (%) | 18 (11.5) | 7 (4.5) |  | 43 (10.4) | 51 (12.3) |  |
| QRS duration, ms | 135.8 ± 8.0 | 142.5 ± 8.0 | 0.098 | 136.1 ± 7.2 | 142.8 ± 6.9 | 0.980 |
| 6MWT | 189.78±26.95 | 252.25 ± 43.57 | 0.001* | 193.24±19.42 | 213.93 ± 25.42 | 0.268 |
|  |  |  |  |  |  |  |
| **Echocardiographic parameters** |  |  |  |  |  |  |
| LVEF (%) | 26.1±8.7 | 39.3±4.6 | 0.001* | 27.0±10.5 | 31.7±4.5 | 0.102 |
| LVEDd (mm) | 65.3±7.6 | 59.9±8.3 | 0.001* | 64.5±6.8 | 62.0±7.1 | 0.070 |
| LVESd (mm) | 41.3±4.9 | 37.5±5.2 | 0.005* | 40.9±5.9 | 39.2±5.9 | 0.495 |
| LVEDv (ml) | 220.1 ± 22.1 | 172.1±27.2 | 0.001* | 217.2 ± 15.6 | 198.7±19.9 | 0.062 |
| LVESv (ml) | 135.5 ± 17.1 | 105.8±33.8 | 0.001* | 133.0±18.4 | 125.4±22.2 | 0.098 |
| Mitral insufficiency  + (%)  ++ (%)  +++ (%) | 58 (37.2)  76 (48.7)  22 (14.1) | 81 (51.9)  66 (42.3)  9 (5.8) | 0.001* | 169 (40.7)  205 (49.4)  41 (9.9) | 125 (30.1)  264 (63.6)  26 (6.2) | 0.001* |
|  |  |  |  |  |  |  |
| **Biomarkers of inflammation** |  |  |  |  |  |  |
| Lymphocytes, n x 10^3^ | 6.94±1.18 | 6.12±1.30 | 0.001* | 7.14±1.79 | 7.97 ± 1.52 | 0.001* |
| Neutrophiles, n | 4.66 ± 0.98 | 5.30 ± 1.38 | 0.001* | 4.74 ± 0.89 | 5.78 ± 1.20 | 0.001* |
| LNR | 1.54±0.37 | 1.21±0.35 | 0.005* | 1.56±0.48 | 1.43±0.39 | 0.001* |
| BNP,(pg/ml) | 341.9±187.8 | 166.52±86.1 | 0.001* | 322.6±201 | 353.6±150.3 | 0.001* |
| NT-proBNP, (pg/ml) | 1836.8±958.6 | 1056.9±108.6 | 0.001* | 1728.7±706.2 | 1468.8±93.6 | 0.001* |
| CRP (mg/L) | 6.09±0.49 | 6.98±0.49 | 0.011* | 5.72±0.39 | 8.93±0.30 | 0.001* |
| Nitrotyrosine (A.U.) | 51.50±4.45 | 36.03±12.49 | 0.001* | 51.10±4.74 | 45.27±11.06 | 0.005* |
|  |  |  |  |  |  |  |
| **Medications** |  |  |  |  |  |  |
| Amiodarone, n (%) | 37 (23.7) | 34 (21.8) | 0.563 | 109 (26.3) | 120 (28.9) | 0.818 |
| ACE inhibitors, n (%) | 58 (37.2) | 51 (32.7) | 0.406 | 184 (44.3) | 168 (40.5) | 0.261 |
| ARS blockers, n (%) | 25 (16) | 21 (13.5) | 0.523 | 81 (19.5) | 68 (16.4) | 0.240 |
| Sacubitril/valsartan, n (%) | 35 (22.4) | 43 (27.6) | 0.296 | 103 (24.8) | 152 (36.6) | 0.001* |
| Beta blockers:  Carvedilol, n (%)  Bisoprolol, n (%) | 72 (46.2)  52 (33.3) | 75 (48.1)  46 (29.5) | 0.734  0.464 | 189 (45.5)  132 (31.8) | 196 (47.2)  133 (32.0) | 0.626  0.941 |
| Aspirin, n (%) | 58 (37.2) | 68 (43.6) | 0.0189 | 178 (42.9) | 197 (47.5) | 0.185 |
| Tiklopidine, n (%) | 5 (3.2) | 9 (5.8) | 0.274 | 18 (4.3) | 25 (6) | 0.273 |
| Warfarin, n (%) | 35 (22.4) | 39 (25) | 0.788 | 90 (21.7) | 109 (26.13) | 0.122 |
| NOAC, n (%) | 48 (30.8) | 49 (31.4) | 0.903 | 108 (26) | 126 (30.4) | 0.165 |
| Calcium antagonist, n (%) | 10 (6.4) | 13 (8.3) | 0.516 | 36 (8.7) | 48 (11.6) | 0.167 |
| Ivabradine, n (%) | 38 (24.4) | 45 (28.8) | 0.238 | 109 (26.3) | 137 (33) | 0.033* |
| Digoxin, n (%) | 60 (38.4) | 55 (35.3) | 0.211 | 148 (35.7) | 151 (36.4) | 0.828 |
| Loop diuretics, n (%) | 134 (85.9) | 128 (82.1) | 0.354 | 364 (87.7) | 381 (91.8) | 0.052 |
| Aldosterone Blockers, n (%) | 91 (58.3) | 84 (53.8) | 0.425 | 235 (56.6) | 253 (61) | 0.204 |
| SGLT2i, n (%) | 25 (16) | 32 (20.5) | 0.305 | 74 (17.8) | 135 (32.5) | 0.001* |
| Statins, n (%) | 115 (73.7) | 120 (76.9) | 0.511 | 308 (74.2) | 331 (79.8) | 0.058 |
| Insulin, n (%) | 37 (23.7) | 44 (28.2) | 0.366 | 95 (20.9) | 101 (24.3) | 0.624 |
| Metformin, n (%) | 88 (56.4) | 91 (58.3) | 0.731 | 246 (59.3) | 256 (61.7) | 0.478 |
| Sulfonylureas, n (%) | 36 (23.1) | 43 (27.6) | 0.362 | 89 (21.4) | 96 (23.1) | 0.559 |
| Thiazolidinediones, n (%) | 17 (10.9) | 20 (12.8) | 0.599 | 50 (12) | 57 (13.7) | 0.468 |
| GLP-1 agonist, n (%) | 26 (16.7) | 29 (18.6) | 0.656 | 69 (16.6) | 81 (19.5) | 0.279 |
| DPP-4 inhibitors, n (%) | 30 (19.2) | 35 (22.4) | 0.486 | 86 (20.7) | 96 (23.1) | 0.402 |

**Supplementary table 2.** The table represents the linear mixed model (LMM) approach. The treatment group (vericiguat users vs non-users) was modeled as a fixed effect, while time was modeled as a random effect to account for within-subject correlation across repeated measurements. We included the intraclass correlation coefficients (ICCs) for the main models, along with a description of the model assumptions and diagnostic checks (including assessment of normality of residuals and homoscedasticity).

| **Parameter** | **Test / Method** | **Result** |
| --- | --- | --- |
| Intraclass Correlation (ICC) | Variance components | 0.37 |
| Normality of residuals | Shapiro–Wilk test | p = 0.18 |
| Homoscedasticity | Breusch–Pagan test | p = 0.29 |
| Linearity | Residual vs fitted plot | No deviation |
| Influence diagnostics | Cook’s distance | < 0.5 for all* |

**Supplementary table 3.** In this table the Poisson regression analysis as a sensitivity analysis to evaluate Cardiac resynchronization therapy (CRT) responders (A) and Heart failure hospitalization (B) rates at 12 months.

1. **Poisson Regression Analysis for CRT Response.**

| **Variable** | **Incidence Rate Ratio (IRR)** | **95% CI** | **p-value** |
| --- | --- | --- | --- |
| Vericiguat | 2.41 | 1.68 – 3.45 | <0.001* |
| Sacubitril/valsartan | 1.38 | 1.04 – 1.89 | 0.026* |
| Baseline ATP production | 0.32 | 0.18 – 0.55 | <0.001* |
| LVEF at baseline | 1.52 | 1.17 – 1.98 | 0.003* |
| Age | 0.98 | 0.96 – 1.01 | 0.142 |
| Male sex | 1.09 | 0.82 – 1.46 | 0.551 |

1. **Poisson Regression Analysis for heart failure hospitalizations at 12 months**

| **Variable** | **Incidence Rate Ratio (IRR)** | **95% CI** | **p-value** |
| --- | --- | --- | --- |
| Vericiguat | 0.34 | 0.22 – 0.51 | <0.001* |
| Sacubitril/valsartan | 0.69 | 0.45 – 0.91 | 0.014* |
| Baseline ATP production | 2.97 | 1.84 – 4.79 | <0.001* |
| LVEF at baseline | 1.41 | 1.10 – 1.88 | 0.009* |
| Age | 1.02 | 0.99 – 1.04 | 0.121 |
| Male sex | 1.11 | 0.87 – 1.43 | 0.386 |

**Supplementary figure 1.**

**
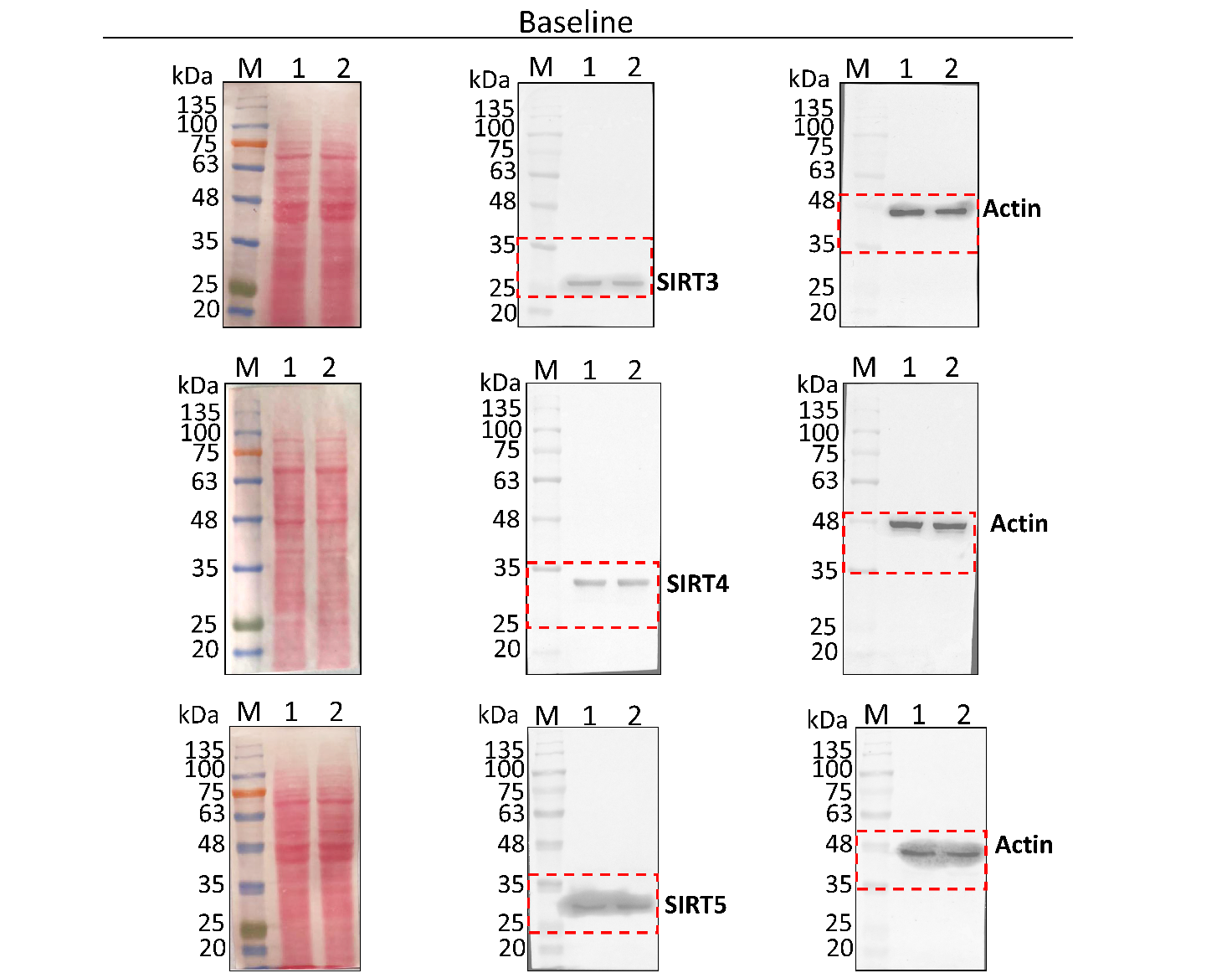
**

**Supplementary figure 2.**

**
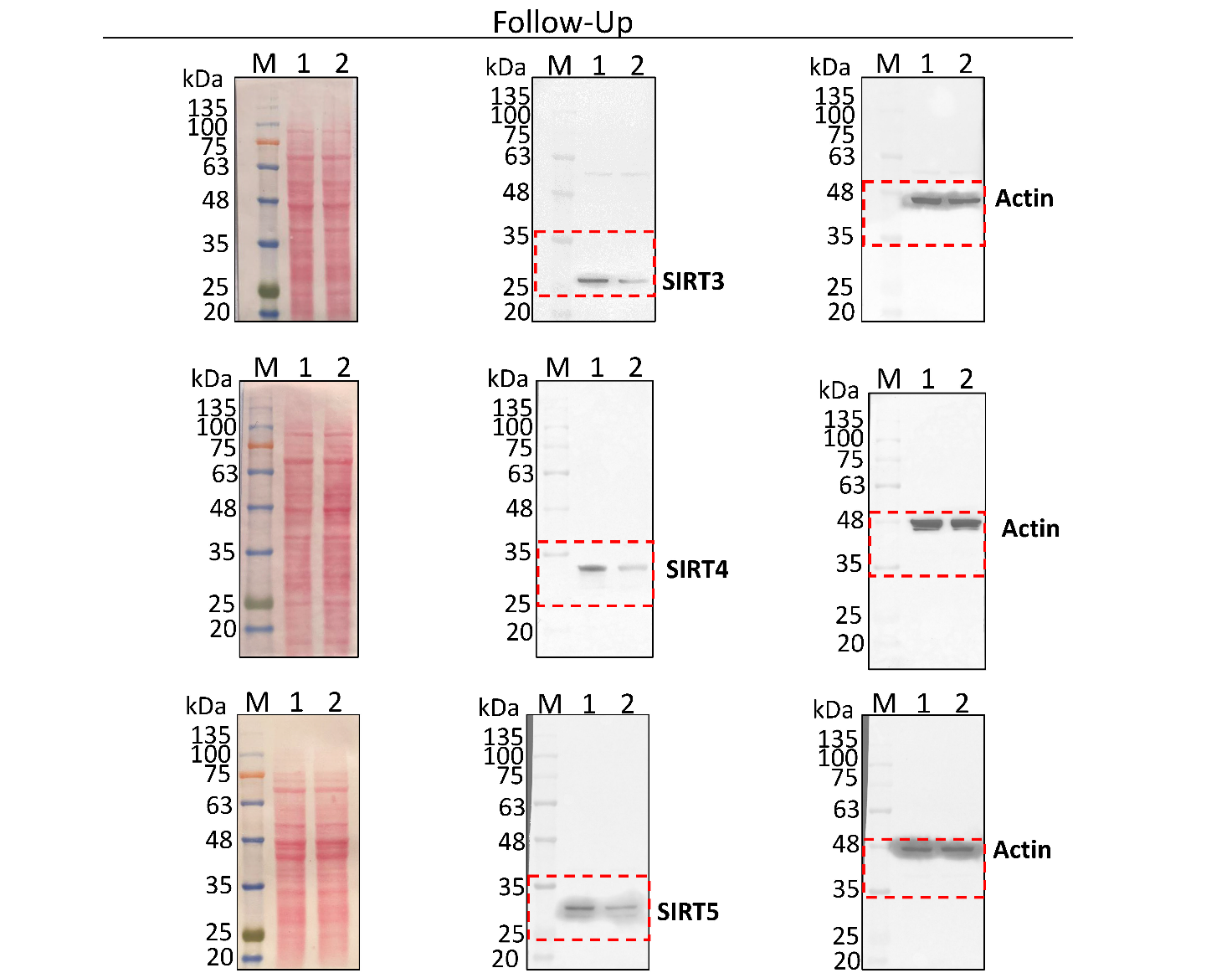
**

**Supplementary figure 3.**

**
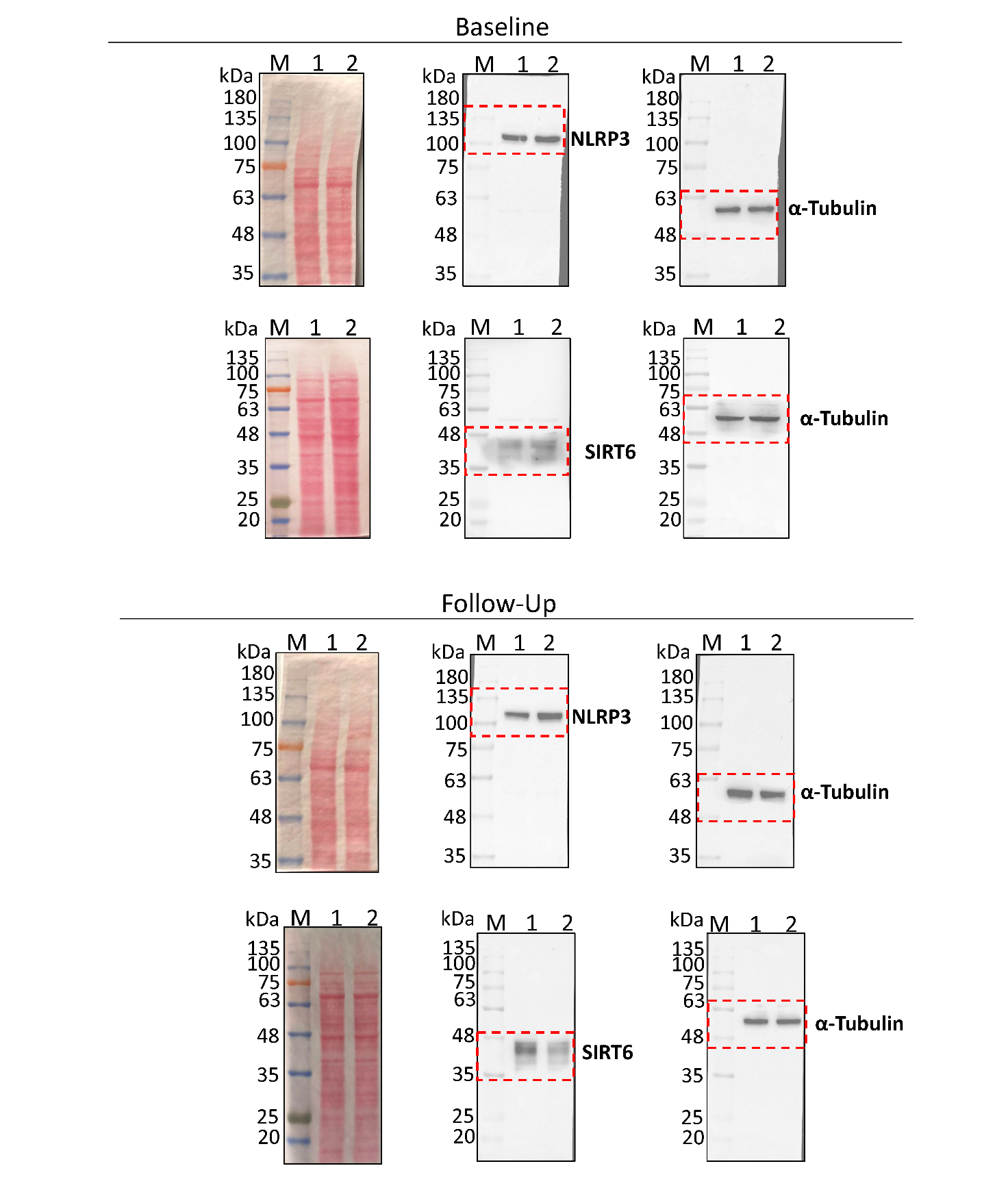
**

STROBE Checklist

| **Item** | **STROBE Section** | **Description** | **Manuscript page** |
| --- | --- | --- | --- |
| 1 | Title and Abstract | Indicated observational cohort design in title/abstract | 1, 3 |
| 2 | Background/Rationale | Scientific background and rationale provided | 5, 6 |
| 3 | Objectives | Specific objectives and hypotheses stated | 5, 6 |
| 4 | Study Design | Retrospective cohort design described | 9 |
| 5 | Setting | Multicenter setting and study period reported | 7, 8 |
| 6 | Participants | Eligibility criteria and selection described | 7, 8 |
| 7 | Variables | Outcomes, exposures, and confounders defined | 7-15 |
| 8 | Data Sources | Clinical records and laboratory data described | 7 |
| 9 | Bias | Potential sources of bias addressed | 7 |
| 10 | Study Size | Sample size explained | 17, 18 |
| 11 | Quantitative Variables | Handling of quantitative variables described | 17, 18 |
| 12 | Statistical Methods | Statistical methods detailed | 17, 18 |
| 13 | Participants Flow | Numbers at each stage reported | 17, 18 |
| 14 | Descriptive Data | Baseline characteristics reported | 19-22 |
| 15 | Outcome Data | Outcome events reported | 19-22 |
| 16 | Main Results | Adjusted and unadjusted estimates reported | 19-22 |
| 17 | Other Analyses | Subgroup and sensitivity analyses described | See supplementary files |
| 18 | Key Results | Key findings summarized | 20, 21 |
| 19 | Limitations | Study limitations discussed | 26, 27 |
| 20 | Interpretation | Results interpreted cautiously | 29 |
| 21 | Generalisability | External validity discussed | 29 |
| 22 | Funding | Sources of funding disclosed | 2 |
